# Preparations, Properties, and Applications of Polyaniline and Polyaniline Thin Films—A Review

**DOI:** 10.3390/polym13122003

**Published:** 2021-06-18

**Authors:** Mahnoush Beygisangchin, Suraya Abdul Rashid, Suhaidi Shafie, Amir Reza Sadrolhosseini, Hong Ngee Lim

**Affiliations:** 1 Material Processing and Technology Laboratory, Institute of Advanced Technology, Universiti Putra Malaysia, Serdang 43400, Selangor, Malaysia; m.beygi2300@yahoo.com; 2 Functional Device Laboratory, Institute of Advanced Technology, Universiti Putra Malaysia, Serdang 43400, Selangor, Malaysia; suhaidi@upm.edu.my (S.S.); hongngee@upm.edu.my (H.N.L.); 3 Faculty of Engineering, Universiti Putra Malaysia, Serdang 43400, Selangor, Malaysia; 4 Magneto-Plasmonic Lab, Laser and Plasma Research Institute, Shahid Beheshti University, Tehran 1983969411, Iran

**Keywords:** polyaniline, PANI, synthesis, thin films, chemical and physical methods, PANI applications, sensors, drug delivery, anti-corrosion

## Abstract

Polyaniline (PANI) is a famous conductive polymer, and it has received tremendous consideration from researchers in the field of nanotechnology for the improvement of sensors, optoelectronic devices, and photonic devices. PANI is doped easily by different acids and dopants because of its easy synthesis and remarkable environmental stability. This review focuses on different preparation processes of PANI thin film by chemical and physical methods. Several features of PANI thin films, such as their magnetic, redox, and antioxidant, anti-corrosion, and electrical and sensing properties, are discussed in this review. PANI is a highly conductive polymer. Given its unique properties, easy synthesis, low cost, and high environmental stability in various applications such as electronics, drugs, and anti-corrosion materials, it has attracted extensive attention. The most important PANI applications are briefly reviewed at the end of this review.

## 1. Introduction

In previous work, researchers focused on producing conductive polymers, which are achieved in the fields of optics, electronics, energy, and so on [1,2,3,4]. Basic types of conductive polymers are obtained through high-precision molecular design and a suitable preparation process [5].

Polyaniline (PANI) was first known as black aniline and came in different forms depending on its oxidation level. Furthermore, PANI is known for its simplicity [6], environmental stability, and ability to be doped by protonic acids [5]. PANI can be determined by connecting 1, 4-coupling of aniline monomer parts. PANI might exist in different oxidation states, and it can be characterized with FTIR benzenoid to quinonoid ratios.

Conjugated conductive polymers principally involve polyaniline (PANI), polythiophene (PTH), polypyrrole (PPY), and their products [7,8]. They have numerous potential applications involving electromagnetic interference shielding, photothermal therapy, rechargeable battery, photovoltaic cell, gas separation membrane, chemical sensor, anti-corrosion coating, microwave absorption, and so on [9,10,11]. Additionally, conductive polymers are utilized, such as conducting fillers in insulating polymer substrates to acquire conducting polymer compounds [12,13,14,15,16]. These compounds possess potential applications in electromagnetic interference shields, electronic equipment, and display device electrodes [17,18,19,20].

Although conductive polymers, particularly PANI, have several unique advantages, they have several disadvantages as well. For example, the biological applications of PANI are limited because of its low processing capacity, inflexibility, and lack of biodegradability. The main problem with PANI is the poor solubility affected by the hard spine [21]. Various methods have attempted to improve its processability, and two substantial attempts to overcome these disadvantages can be chemical modifications, namely, doped PANI and alternative PANI derivatives. The chemically modified PANI not only demonstrates improved processing capability, but enhanced conductivity and anti-corrosion properties compared with pure PANI.

In general, the preparation of PANI-based compounds with organic and inorganic nanofillers is believed to be a potential route to improve the properties and performance of PANI. Such approaches result in materials with synergistic or complementary features between PANI and organic/inorganic nanoparticles. Organic polymers that possess the magnetic, optical, and electric features of metals are called intrinsically conducting polymers, and such polymers are the electroactive polymers that possess the behaviors mentioned above while retaining their structural features. These polymers have conjugated double bonds in their backbones and determine good electrical conductivity without the use of conductive additives. They convert to high conductivity alone in a doped state. The conductivity of the polymers is improved to a metallic state from their insulating state through the doping process whereby both N-type (electron donors) and P-type (accepting electrons) dopants are used to induce an insulator-to-metal transition in electronic polymers [22,23]. It was shown to be chemically or electrochemically added to or removed from the polymer chain by introducing acidic or basic solutions during polymerization or post-processing of the polymer [24]. During this process, the negative (electron) or positive (hole) ions formed are free and can flow along the polymer chain. Different types of PANI composites with inorganic nanoparticles such as CeO_2_ [25], TiO_2_ [26], ZrO_2_ [27], Fe_2_O_3_ [28], and Fe_3_O_4_ [29] have been described to create materials with the synergistic effect of both PANI and inorganic nanoparticles. These composites are utilized in several fields, such as electrochromic devices, LEDs, EMI shielding, electrostatic discharge systems, batteries, and chemical and biochemical sensors [30].

PANI is found in one of the three idealized oxidation states during the polymerization of aniline monomer: (a) leucoemeraldine (white/clear), (b) emeraldine (salt-green/base-blue), and (c) pernigraniline (blue/violet). Fully oxidized PANI is known as pernigraniline base. Half of the oxidized PANI is reduced as the emerald base, and PANI is completely reduced as the lecomeraldine base [31]. Among these, emeraldine is the most stable and conductive, with a conductivity in the range of 10−10 s cm^−1^, while its salt form has a conductivity of 30 s cm^−1^. The exact conductivity of PANI depends on the synthesis method, and it can be controlled by submerging the emeraldine base in an aqueous acidic solution of picric, phosphoric, or camphor sulfonic acid [32]. The emeraldine base form of PANI is difficult to process because of the lack of solubility, and it is obtained as a dark powder. The lack of solubility is due to the stiff polymer backbone and the hydrogen bonding interaction between adjacent chains. In addition, the emeraldine base form of PANI is unstable at a melt processing temperature, which restricts its industrial applications.

**Figure 1:**
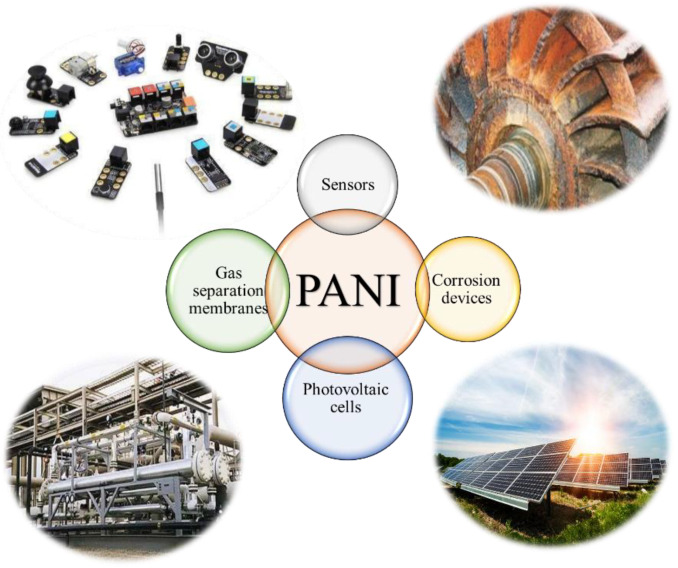
Polyaniline in different applications.

PANI has potential applications in various fields, such as organic electronics, biomedical fields, and anti-corrosion materials due to its extraordinary properties, such as exceptional electrical features, decent chemical and environmental stability, low cost, and easy preparation process [33]. It has several applications, such as chemical sensors [34,35], corrosion devices [36], photovoltaic cells [37], and gas separation membranes [38], as shown in Figure 1. In addition to all the probable applications detailed above, PANI microtubes/nanofiber and PANI-multi-walled carbon nanotubes [39] and nanocomposites are used as microwave safeguards and electromagnetic shielding materials [40].

This study principally focuses on advances in PANI chemical modification and advanced features over the past decades, which serve as a strategic guide to establish a nearby link between PANI chemical modifications and practical applications. In addition, the review deals with the preparation of PANI and PANI thin films by several methods to provide a background for future research.

### 1.1. History of Polyaniline Development

In 1977, Shirakawa [41] reported that polystyrene was doped in place of a conductive polymer. They found that only conductive polymers were steady enough under normal processing circumstances. As a major candidate, PANI has several unique features compared to other conductive polymers, such as polythene, polypyrrole, polyphenylene, polyphenylene vinylene, and polystyrene. PANI possesses good thermal resistance, so it is easily prepared by chemical and electrochemical methods in different organic solvents or even in aqueous media [42]. In addition, PANI has gained increasing attention due to its low price, good environmental stability, excellent optical and electrical properties, and good anti-corrosion features that ensure its widespread use in commercial and technological applications [43]. These applications include secondary batteries [44], electromagnetic interference protector [45], solar cells [46], biology or chemical sensors [47], corrosion devices [48], organic light-emitting diodes [49], and electrorheological materials [50].

**Figure 2:**
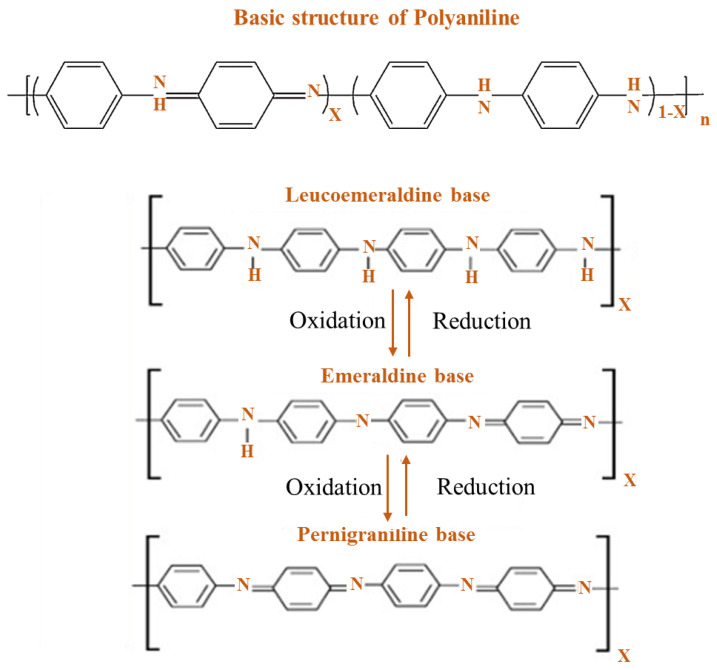
Basic PANI in three dissimilar types (0 ≤ x ≤ 1). Reproduced from Prog. Polym. Sci, Vol 23, Gospodinova, N.; Terlemezyan, L., Conducting polymers prepared by oxidative polymerization: Polyaniline, pp. 1443–1484, Copyright (1998), with permission from Elsevier.

PANI is produced in one of three perfect oxidation forms during polymerization of aniline monomer: (a) leucoemeraldine, (b) emeraldine (salt/base), and (c) pernigraniline. PANI is present in two different forms: (i) completely reduced form, which contains only benzenoid rings, and (ii) fully oxidized form containing benzenoid and quinonoid ring as repeating units [51,52]. In 1997, MacDiarmid first suggested dissimilar forms for pure PANI [53]: entirely reduced leucomeraldin or LEB (x = 0), partial-oxidized emerald or EB (x = 0.5), and entirely oxidized peringranillin or PAB (x = 1) (Figure 2). Among them, proton acid doping can convert EB to a conductor; nevertheless, LEB and PAB cannot. Hence, EB is the most common type of PANI. PANI and its formatives can be converted from their insulating state to conductive material. Doping methods include the doping of chemicals by charge transfer groups, doping of electrochemical by proton acid, and doping of photo by injecting charge in a metallic/semiconductor polymer interface [54,55,56]. Nevertheless, similar to additional π-bonded polymers, the use for neat PANI can be quite narrow due to their processing and poor solubility [57]. Poor solubility of PANI is due to the presence of a strongly conjugated π electron structure and the high stiffness of the PANI molecular backbone. Thus, two methods were tested to improve its processability and solubility. First, functional proton acids are used in the PANI protein. Second, alternative PANI derivatives are used to enhance their properties.

### 1.2. Advantages of PANI

PANI is the second most widely used electrically conductive polymer after PPy. Chemical and electrochemical methods are employed in the synthesis of PANI, although electrochemical deposition is often preferred because it creates a high purity coating on the surface of the distributed film [58].

**Figure 3:**
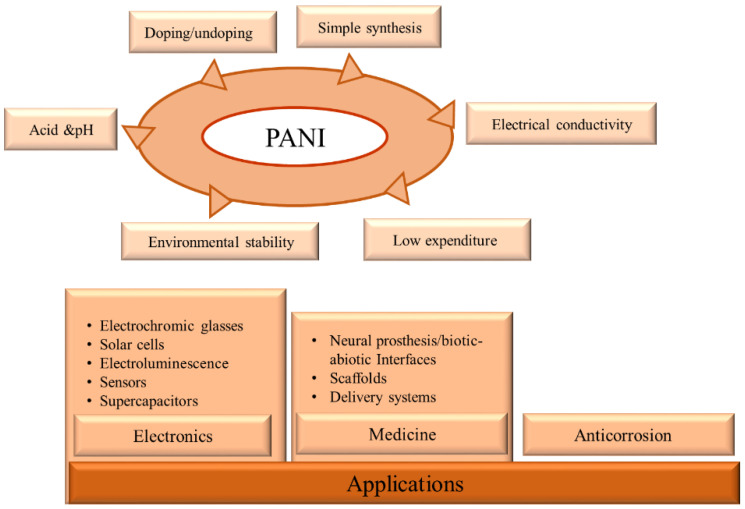
Prominent properties and usage of polyaniline.

Amongst all intrinsic conducting polymers (ICPs), PANI has attracted the attention of many researchers due to its reversible doping/dead doping properties, remarkable electrical conductivity, pH change properties, low expenditure, simple synthesis, and environmental stability [59]. It also has the unique ability to be doped by proton acids (proton doping) apart from conventional redox doping. PANI, through its molecular self-assembly, often forms super-molecular nanofibers, lending itself to a variety of applications due to completely different and new properties because of the high surface-to-volume ratio. Several PANI nanostructures such as nanofibers, nanotubes, and nanospheres have been developed by a set of synthesis methods [60]. The introduction of a secondary component, such as nanomaterials, in PANI, further expands its performance, providing efficient designs and advanced performance. Cooperation between individual components enhances the properties of the nanocomposite and expands its scope of application [11]. The development of PANI nanostructures and their nanocomposites stems from the desire to discover the full potential of these materials. Figure 3 shows the prominent properties and remarkable usage of PANI.

PANI and PANI compounds have been prepared using chemical and physical techniques. The following section discusses the chemical and physical preparation of PANI and PANI compounds.

## 2. Preparation of PANI

**Figure 4:**
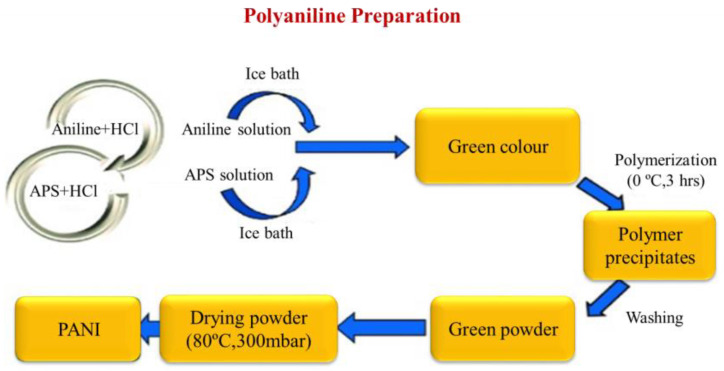
Oxidative polymerization method of polyaniline. Reproduced from Results Phys, vol 14, Fayzan, M.; Nawaz, A.; Khan, R.; Javed, S.; Tariq, A.; Azeem, M.; Riaz, A.; Shafqat, A.; Cheema, H.M.; Aftab, M.; et al., Results in Physics EMI shielding properties of polymer blends with inclusion of graphene nano platelets, 102365, Copyright (2019), with permission from Elsevier.

In recent studies, there have been numerous reports for the oxidative polymerization technique of PANI preparation, as shown in Figure 4. In this technique, polymerization and doping occur simultaneously with chemical or electrochemical methods. In this literature, the chemical method has a higher performance than the electrochemical method [61,62].

### 2.1. Chemical Preparation (Oxidative Polymerization)

**Figure 5:**
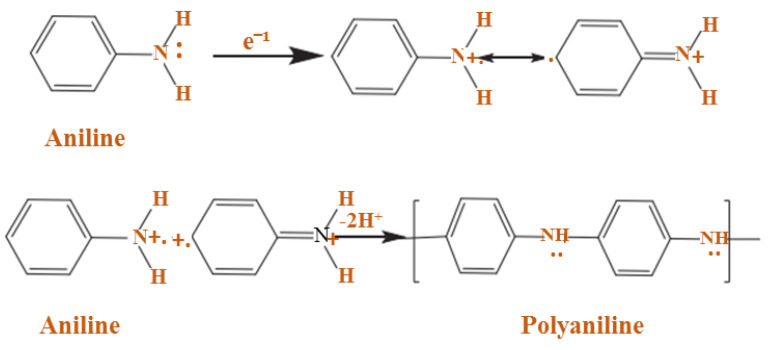
PANI in homo-polymerization. Reproduced with permission from Tan, S.; Zhai, J.; Xue, B.; Wan, M.; Meng, Q.; Li, Y.; Jiang, L.; Zhu, D. Property Influence of Polyanilines on Photovoltaic Behaviors of Dye-Sensitized Solar Cells, Langmuir 2004, 20, 2934–2937. American Chemical Society.

In chemical oxidation polymerization, PANI is synthesized by using hydrochloric acid (HCl) or sulfuric acid (H_2_SO_4_) as a dopant and ammonium persulfate (APS) as an oxidant in an aqueous environment [63,64]. At this time, a proton can be removed by the oxidant from the monomer of aniline without either creating a heavy bond, or with the absolute product (Figure 5).

In chemical synthesis, there are three reactants of PANI that require aniline, oxidant, and acidic medium. HCl and H_2_SO_4_ are common acids used in the synthesis of PANI, whereas ammonium sulfate ((NH_4_)_2_S_2_O_8_), hydrogen peroxide (H_2_O_2_), sodium vanadate (NaVO_3_), cerium sulfate (Ce(SO_4_)_2_), Potassium dichromate (K_2_Cr_2_O_7_), potassium iodate (KIO_3_), and potassium free cyanide (K_3_ (iron (CN)_6_) can be used as an oxidizer. The oxidant polymerization method is the most communal mechanism used to synthesize PANI, while aniline reacts with an acidic substance as a neutralizing agent and polymerizes by adding one drop of an oxidizing agent such as ammonium persulfate (APS) at dissimilar temperatures. After realization, the polymerization mechanism (3 h) was an aqueous solution separated by filtration. Pure PANI can be obtained by rinsing the above solution 5–6 times with deionized water. Alcohol and acetone are then added to make the filter colorless and ensure that non-reactive materials are completely removed. The product is slime green and is well-known as PANI polymeraldine salt, which is unstable due to the existence of swords. Hence, the polymeraldine salt converts to the PANI-EB structure, which is naturally stable at room temperature, allowing this precipitate to equilibrate with an appropriate amount of NH_4_OH [24].

Camphor sulfonic acid (CSA) with thin films of PANI doped on simple glass is prepared with the chemical polymerization method and rotated with a spin coater [65]. The adhesion efficacy of the thin film to the glass is excellent because of organic solvent and unpleasant organic matter, whereas the FTIR results showed the existence of dopant and change in their molecular structure for the thin film by an extraordinary CSA extraction fraction (1:8). These pristine films have an amorphous nature and have been shown to change with the increasing doping ratio of CSA to the crystalline structure. SEM shows a variation in smooth morphology to rough morphology, such as a root of PANI thin films.

### 2.2. Electrochemical Preparation

Electrochemical preparation of PANI focuses on the electrochemical method and electrochemistry. Hence, this is an electro-organic method compared with organic preparation. Electrochemistry shows a major role for conductive polymers (CPs) in preparation because, in most applications, the synthesis of polymers such as a thin film by a sharp structure that has an outsized surface area is essential [66,67]. When CP is synthesized through the chemical method, then it cannot easily proceed with the electrochemical process because these thin layers follow the surface electrode that is utilized in the study of optical properties and electricity. The electrochemical preparation method is similar to the electro-deposition method of metals in which CP is deposited on an electrode surface. Potentiodynamic and galvanostatic techniques have been utilized for PANI preparation as electrochemical methods. The electrochemical preparation method includes numerous benefits over the chemical method: (1) cheap and simple method; (2) doping nature of the polymer solution will continue with the desired ion by changing; (3) a catalyst cannot require a chemical method in the electrochemical method; (4) homogeneous and pure are essential features of CPs, which are then deposited on the electrode surface; (5) the electrochemical synthesis is completed in a chamber cell that contains the electrode–electrolyte and a power source.

**Figure 6:**
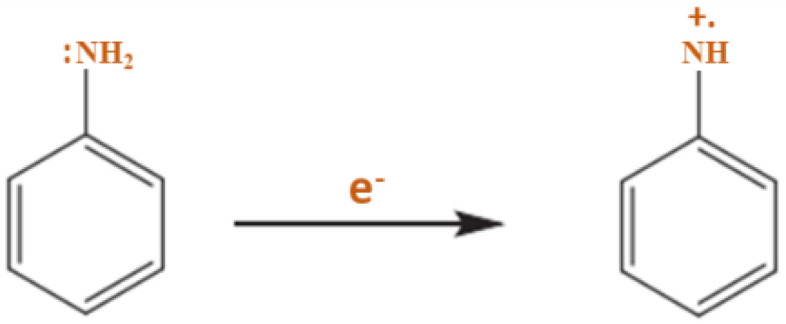
Structure of radical cation of aniline. Reproduced with permission from J. Electroanal. Chem.,Vol 236 Genies, E.M.; Lapkowski, M. Spectroelectrochemical evidence for an intermediate in the electropolymerization of aniline, pp. 189–197. Copyright (1987), with permission from Elsevier.

**Figure 7:**
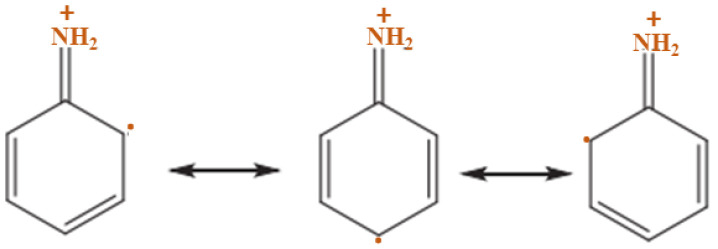
Aniline radical cation in the resonating form. Reproduced with permission from Materials Chemistry and Physics, Vol 69, Koval, E.P.; Whittingham, S.; Skolozdra, O.M.; Zavalij, P.Y.; Zavaliy, I.Y.; Reshetnyak, O.V; Seledets, M. Co-polymers of aniline and nitroanilines. Part I. Mechanism of aniline oxidation polycondensation, pp. 154–162. Copyright (2001), with permission from Elsevier.

**Figure 8:**
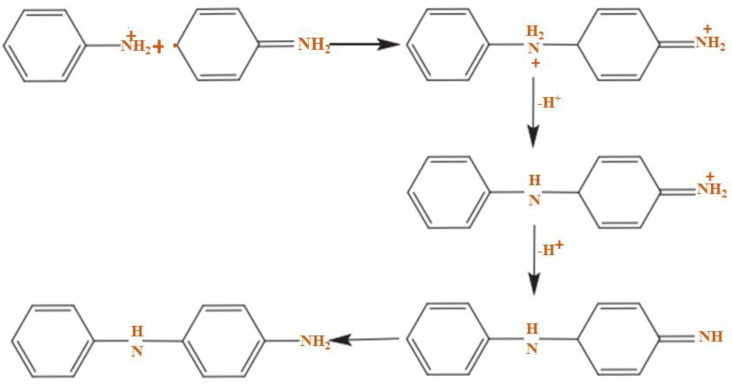
Structure for the dimer. Reproduced with permission from Materials Chemistry and Physics, Vol 69, Koval, E.P.; Whittingham, S.; Skolozdra, O.M.; Zavalij, P.Y.; Zavaliy, I.Y.; Reshetnyak, O.V; Seledets, M. Co-polymers of aniline and nitroanilines. Part I. Mechanism of aniline oxidation polycondensation, pp. 154–162, Copyright (2001), with permission from Elsevier.

**Figure 9:**
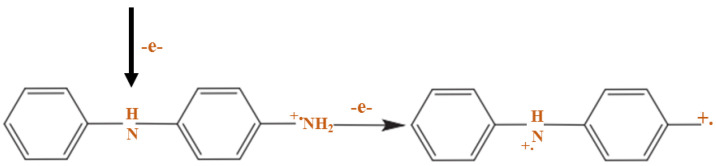
Structure for the radical cation of dimer. Reproduced with permission from Materials Chemistry and Physics, Vol 69, Koval, E.P.; Whittingham, S.; Skolozdra, O.M.; Zavalij, P.Y.; Zavaliy, I.Y.; Reshetnyak, O.V; Seledets, M. Co-polymers of aniline and nitroanilines. Part I. Mechanism of aniline oxidation polycondensation, pp. 154–162, Copyright (2001), with permission from Elsevier.

**Figure 10:**
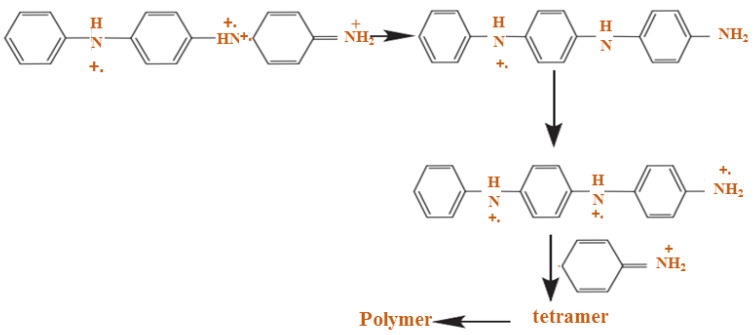
Structure of polymer preparation. Reproduced from Eur. Polym. J., 125, Kumari Jangid, N.; Jadoun, S.; Kaur, N., A review on high-throughput synthesis, deposition of thin films, and properties of polyaniline, Copyright (2020), with permission from Elsevier.

Furthermore, the PANI polymerization method can be performed in three steps. The first step of the polymerization synthesis mechanism is the oxidation step, in which aniline is converted to only nitrogen by removing an electron from the couple in the radical cation (Figure 6). Secondly, a catalyst during the process will be accelerated. The cationic radical aniline can be signified by three resonant structures (Figure 7); between the three forms of resonance, the second is more reactive than the first due to an alternative induction effect and the lack of a stere barrier. Thirdly, the reaction is accomplished among the radical cation and the next resonant of the aniline radical cation in top on the tail manner; then dimer can be made (Figure 8) [68,69]. Finally, the electrons are eliminated and converted to a different radical cation, which can be oxidized by the dimer (Figure 9). Radical cation mostly responds by the radical cation monomer or the radical cation dimer to a trimer or tetramer and polymer, respectively, as presented in Figure 10 [68].

### 2.3. Doping of PANI

**Figure 11:**
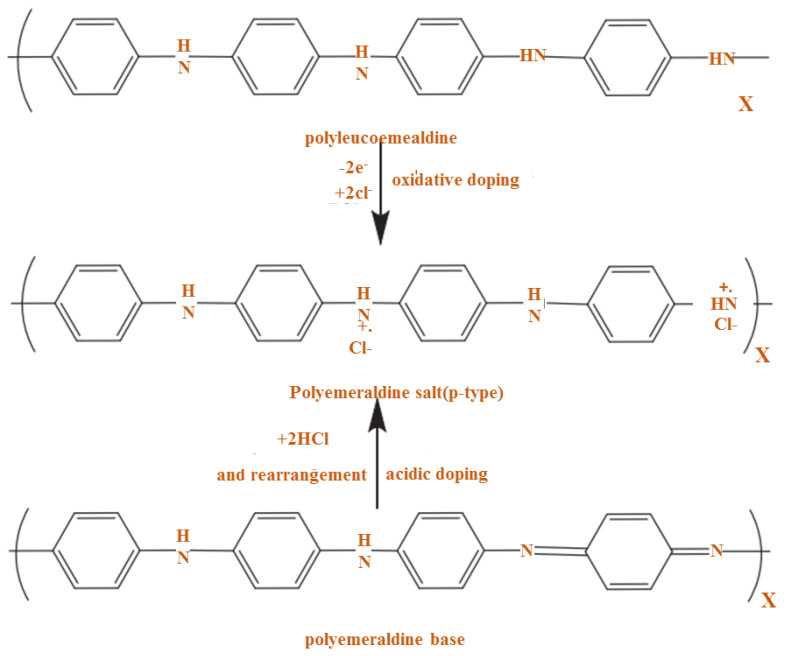
Structure of doping in PANI.

Here, doping is used to indicate similarities with some semiconductors such as silicon (Si) or germanium (Ge) in that P or B atoms are presented. Doping in the CP causes oxidation or reduction, so the polymers can be considered p and n-type, respectively. Given the conduction mechanism of PANI, which is the polymer-based proton or poly-alkomeral oxidation, it is a prominent conductive polymer. In this process, PANI is converted to the polymeraldine salt form shown in Figure 11 [70].

### 2.4. Oxidative Doping

Oxidative doping of PANI should be performed with iodine (I_2_) in CCl_4_ solution, Cl_2_ solution, and FeCl_3_ solution or organic Tin tetrachloride (SnCl_4_) and using O_2_ or H_2_O_2_ in acid solution.

**Figure 12:**
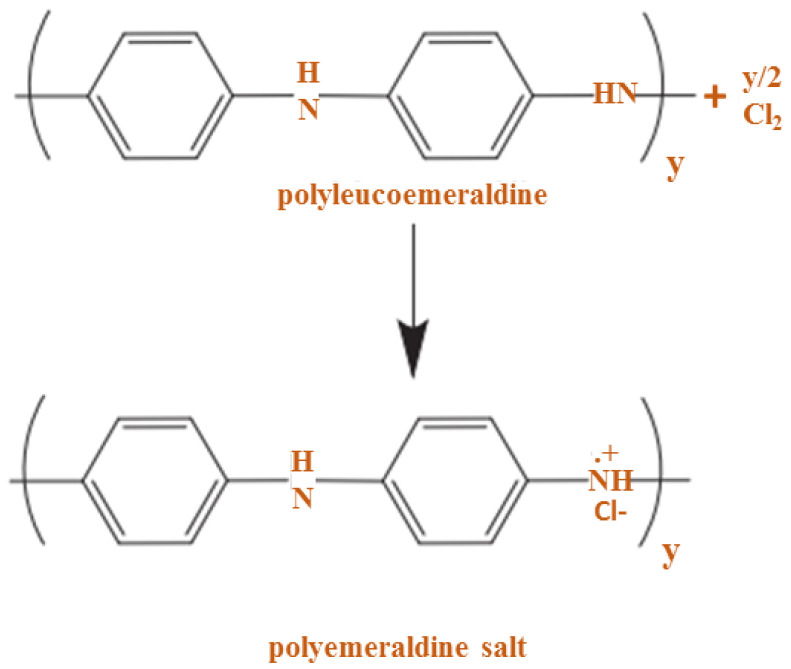
Doping oxidative with chlorine. Reproduced from Eur. Polym. J., 125, Kumari Jangid, N.; Jadoun, S.; Kaur, N., A review on high-throughput synthesis, deposition of thin films, and properties of polyaniline, Copyright (2020), with permission from Elsevier.

**Figure 13:**
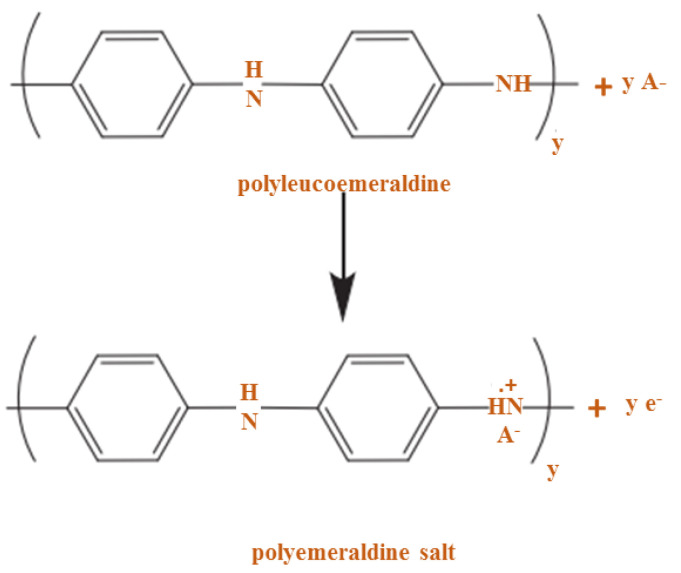
Doping electrochemical of polyleucoemeraldine. Reproduced from Eur. Polym. J., 125, Kumari Jangid, N.; Jadoun, S.; Kaur, N., A review on high-throughput synthesis, deposition of thin films, and properties of polyaniline, Copyright (2020), with permission from Elsevier.

PANI oxidative doping using chlorine molecule is given in Figure 12, where chlorine is considered a dopant and oxidant. Controlling δ doping is a challenging method, even though chemical doping is an efficient procedure. The challenge should be solved by electrochemical doping, which is determined by a voltage applied among the counter electrode and the CP [71]. The polyalkoeramedrine base should be written as shown in Figure 13.

### 2.5. Acidic Doping

The existence of the dopant is significant because the charge carriers and the conductivity of the polymer can be affected by the grade and type of dopant used. Two forms of redox doping can be used in the process of producing a conductive polyaniline polymer: the first is n-type doping (reduction) and the second is p-type doping (oxidation) [72,73,74]. A radical cation is made during polymerization development by removing an electron from the structure of a monomer. Any radicals that are produced will be joined together to form a monomer chain. The following equation shows a simple reaction that demonstrates chemical polymerization [75];

$$\mathrm{Monomer}\;+\;\mathrm{dopants}\overset{\;\mathrm{Oxidant}\;\left(0-1\;{}^{\circ}C\right)\;}{\to }\mathrm{polymer}$$

Classically, hydrochloric acid (HCl), sulfuric acid (H_2_SO_4_), and chloric acid (HClO_3_) are used as dopants in conductive PANI synthesis. In acid doping, the basic action of polymeraldine with HCl, the H_2_SO_4_ acids that induce protons at immune spots and give polymeraldine salt, follows through the mechanism shown in Figure 12. One weakness of polymeraldin hydrochloride salt is a poor solution in ordinary solvents, and conductivity varies by temperature [76]. Hence, polymeric acid such as polyacrylic acid can be obtained as a drug in hydrochloride solution to improve solubility and temperature stability [77,78].

## 3. Synthesis of Thin Films of PANI with Different Methods

**Figure 14:**
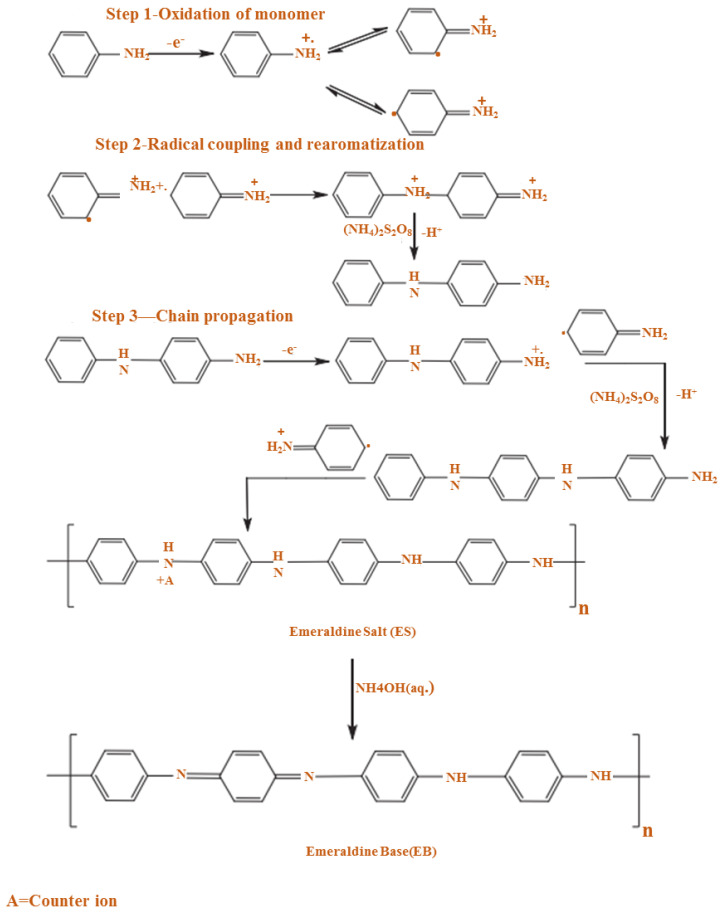
PANI with oxidative polymerization of aniline. Reproduced from Prog. Polym. Sci., Vol 23, Gospodinova, N.; Terlemezyan, L., Conducting polymers prepared by oxidative polymerization: Polyaniline, pp. 1443–1484, Copyright (1998), with permission from Elsevier.

**Figure 15:**
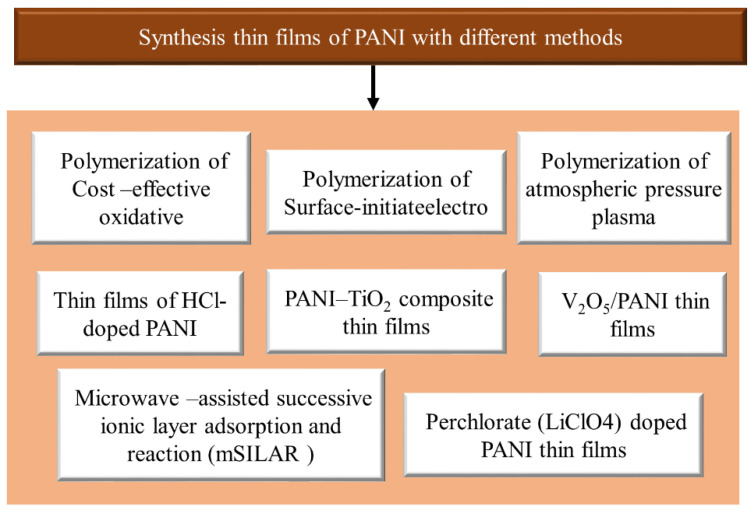
Synthesis of thin films of PANI via different methods.

Preparation of PANI thin films can be deposited on several substrates with chemical and physical techniques. The chemical technique is categorized into three forms: bulk oxidative chemical polymerization, localized surface polymerization, and chemical vapor deposition (CVD). Equally, the physical method is essentially concerned with the type of electrodeposition. These unique synthetic methods permit PANI to show changed physicochemical features and similarly allow improved control of electrical conductivity, environmental stability, and thermal stability.PANI due to synthetic oxidative polymerization of aniline (Figure 14) is created by adding a drop of ammonium persulfate (APS) that is utilized as an oxidizing agent at several temperatures and constant stirring for 3 h in the existence of an acidic medium as a dopant [51]. After the polymerization mechanism (3 h), a blue solution is perceived that is detached with filtration. PANI should be achieved by washing the solution five times with distilled water, alcohol, and (CH_3_)_2_CO to decolorize the filter, which ensures the complete removal of inactivated material. This product is dark green as PANI polymeraldine salt, which is unstable because of the existence of anti-stability ions. With this form, the polymeraldine salt converts to the PANI-EB structure, which is naturally stable at ambient temperature, allowing this precipitate to equilibrate with an appropriate amount of NH_4_OH [79]. In this review, the preparation of thin films of PANI via several methods is categorized and shown in Figure 15.

### 3.1. Cost-Effectiveness of Oxidative Polymerization

Thin films of PANI should be prepared by inorganic acids and then deposited on the glass layers through several cost-effective methods [80,81]. The cleaned glass substrate is placed vertically in the mixed reactants for 1 h to announce the PANI film [82]. The thickness of the thin film is 220 nm [83].

### 3.2. Polymerization of Surface-Initiated Electrons

Thin films of PANI are coated on the gold (Au) electrode using the surface of electrochemical polymerization. This technique begins with an alternative to 4-nitrobenzenediazoniumsalt (NBDS) or the formation of a layer of 4-aminophenol (ATP) by reducing nitrogen dioxide (NO_2_) as a functional group in an aniline monolayer. Hence, a bond is made on the surface obtained by a well-organized PANI structure. A thin film of PANI demonstrates a peak that distinguishes the redox shift amongst reduced and oxidized forms because of a change in direction to negative potential [84].

### 3.3. Polymerization of Atmospheric Pressure Plasma

IPC-APPJ can synthesize plasma polymerization of aniline nanofiber and nanoparticles. In this technique, the IPC-APPJ works with the package, which includes three glass pipes to raise plasma jets in disintegration areas. A plastic pipe and a bottom cap of polytetrafluoroethylene (PTFE) are fixed at the end of a jet for capturing the high-density plasma. As a result, the IPC-APPJ can extend to the downstream end and produce a wide and deep glow plasma at disjointed areas with a low cap [85,86].

### 3.4. Microwave-Assisted Successive Ionic Layer Adsorption and Reaction (mSILAR)

Zinc oxide (ZnO) nanocrystalline thin film is regulated by the mSILAR underground framework. ZnO/PANI is fabricated by situ polymerization, and thin films are fabricated by rotating coating. ZnO thin film is launched by mSILAR, which is first fixed on the glass substrate and then replaced in sodium zinc in hot water at 90–95 °C and room temperature using a micro immersion coating control unit and dropping anhydrous aluminum chloride (AlCl_3_) to perform aluminum doping to the shower over sodium [87]. The doping center is kept from a large number of mixtures at 3–5wt%. Clean substrates are immersed for 10 s following immersion in high temperature of the water using proportional variety, and the strategy is repeated. These methods are tempered using an air annealing chamber at 450 °C [88,89].

### 3.5. PANI–TiO_2_ Composite Thin Films

Thin films are prepared by aniline of PANI-TiO_2_ compound, APS solutions, and TiCl_3_. Firstly, a TiO_2_ solution is prepared from a five-day-old TiCl_3_ solution. On the first day, the solution is seen to be purple. On the fifth day, white tubercles are formed, which convert to TiO_2_ in six days. TiO_2_ cell is preserved by the solution of aniline (0.1 M) and H_2_SO_4_ (1 mL) (1:1), and the stainless layers are absorbed in the reaction vessel vertically. APS (0.1 mM) as an oxidizer is then added. In this process, the blue solution turns dark blue and eventually green. Precipitation occurs after 30 min and a thickness of 0.03 mg/cm^2^ is placed on a steel layer at the bottom [90].

### 3.6. Perchlorate (LiClO_4_)-Doped PANI Thin Films

PANI is produced by oxidative polymerization, where it begins with HCl and then LiClO_4_ is added via doping. PANI thin films and their special doping variety are placed on simple glass and ITO glass with a combined bath declaration technique. PANI base is obtained by filtered and dry chemical course methods. Thus, PANI is crushed in N-methyl pyrrolidine (NMP) dissolved in 3 t by weight—a significant amount for a solution. ITO glass and simple glass slides are observed for 72 h. The PANI detached coating obtained by this method is washed, cleaned, and dried. The LiClO_4_ doping period is fabricated in dimethyl carbonate (DMC) by dissolving it at rates of 1%, 2%, and 5%. PANI basic non-attached films are immersed in a specific path of lithium salt for 48 h. These thin films are dried in the oven for 6 h [91].

### 3.7. Thin Films of HCl-Doped PANI

PANI with HCl can be prepared with the chemical oxidative polymerization method by aniline, dopant (such as HCl), and an oxidant (e.g., APS). Hence, APS is added dropwise until polymerization is completed for 15 min, after which a thin film of PANI-HCl is formed. This procedure is repeated at 4 °C, 13 °C, and 31 °C with 1 and 2 M HCl. The synthesized thin layers are washed by distilled water or acetone and then dried at ambient temperature [92].

### 3.8. V_2_O_5_/PANI Thin Films

V_2_O_5_/PANI is used to detect NH_3_ gas in the presence of aerogel V_2_O_5_. In this method, gel reacts with the aniline monomer to form a composite. The V_2_O_5_/PANI thin films without any other gas interference can detect ammonia gas. Aniline and V_2_O_5_ lotions demonstrate an acid–base and redox reaction in polymerization. V_2_O_5_/PANI is formed by reaction with aniline (4.0 mL of 0.2 mol/L) and 30 mL of V_2_O_5_ lotion under continuous stirring at 350 rpm for two days. The product is green, which is coated on a glass and then evaporated at ambient temperature. The resulting thin film is washed by distilled water and then dried [93].

## 4. Deposition of Thin Films of PANI

**Figure 16:**
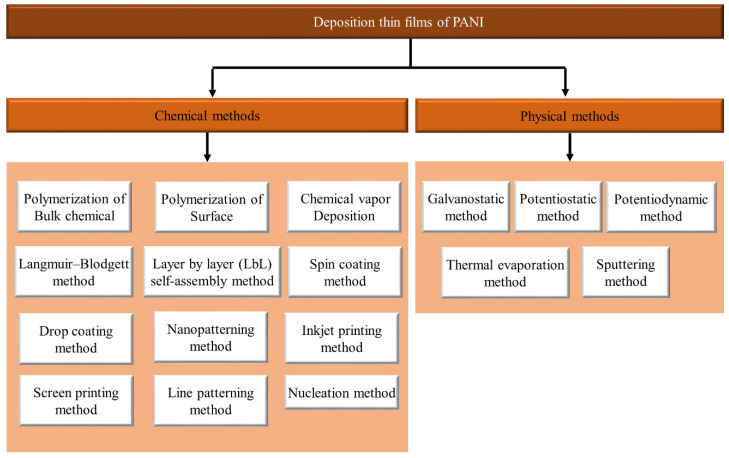
Different methods of thin-film deposition of PANI.

Several methods can be adopted for the deposition of thin films of PANI. As shown in Figure 16, these techniques include chemical and physical methods.

### 4.1. Chemical Techniques

The coating on different materials with advanced electrical polymers (CEPs), such as PANI and their subordinates, is rapidly discovered using composite polymerization methods. This review considers PANI chemical deposition techniques, such as polymerization of bulk chemical, polymerization of surface, chemical vapor deposition, Langmuir–Blodgett method, layer by layer (LbL) self-assembly method, spin coating method, drop coating method, nanopatterning method, inkjet printing method, screen printing method, line patterning method, and nucleation method.

#### 4.1.1. Polymerization of Bulk Chemical

The oxidative polymerization produced regularly follows an ion change during polymerization. Initially, it was a colorless solution that turned blue and dark blue, indicating that PANI dimers and oligomers were formed. This method has a prominent property, in which rapid staining is performed, and the polymerization continues during a certain induction period. The speed of the method depends on several factors, such as the nature of the oxidant and the concentration of the reactant [94]. Most resulting CEPs can be deposited, such as a thin film of different layers with the reaction medium. Different CEPs show several oxidation states. Hence, there is conductivity according to their shape. By removing an electron from the organization by anion doping, the polaronic form of half the oxide is obtained. Similarly, by removing the second electron, an oxide dipole form is produced. Consequently, the PANI-EB formula demonstrates the highest conductivity between PANI-LE and PANI-PB.

#### 4.1.2. Surface Polymerization

Printed circuit boards (PCBs) have been the essential practical application in CEP deposition on the surface for non-conductive substrates with chemical polymerization since the 1990s. The surface of the PCB responds with the bases MnO_2_ and KMnO_4_, which are spread on selected PCB plastic chunks. PANI thin film is deposited on the PCB and then reacted with the PANI acidic solution, where MnO_2_ initiates PANI polymerization. The Cu metal coating precipitates are electrically displaced on the PANI-deposited PCB. Hence, this method has received much consideration, and several materials are involved in this method [95].

#### 4.1.3. Chemical Vapor Deposition

The deposition of initial chemical vapor (iCVD) and deposition of oxidative chemical vapor (oCVD) are capable of depositing coating films on bulk, micro, and nanoscale materials. In the vapor phase of the polymer, many effects of deposition have been observed, such as dissolution and wetting. The deposition rate of iCVD is increased by a warm string of CVD to preserve the chemical nature of the polymers, and an unheated primer is utilized in the stream. Otherwise, in oCVD, it is formed using direct oxidants and monomers into electrically conductive reactors and combustible films. In iCVD, the polymer monomer and the initiator molecule are inserted in the vacuum chamber. Thus, in the iCVD method, several products free of random copolymers, radical homopolymers, and alternating copolymers are produced. The iCVD function is characterized by being superhydrophobic, hydrophilic, antimicrobial, chemical-resistant, biomass, and peptide of polymer surfaces. In oCVD, stepwise growth polymerization of polymers occurs using volatile monomers and oxidants [96]. Hence, adhesive CP coatings exhibit various properties, as demonstrated by oCVD performance [97]. This method creates a covalent bond between the films and the substrate [98]. Advanced adhesion is essential for several practical applications and allows the creation of an extraordinary resolution pattern for the CVD polymer layer [99].

#### 4.1.4. Langmuir–Blodgett Method

The composite thin film of PANI-CSA is prepared by using this method. Hence, the PANI-CSA solution (800 mL) is spread on the material by syringe. Firstly, CHCl3 evaporates then, the monolayer of films completely removes the solvent particles for 20 min. Thus, the gas phase of the film is acquired. Thin films of CdS and PANI-CSA are prepared similarly, with the isotherm checking procedure. In the end, the changes in both of them can be compared through the deposition method. Deposition of LB film is carried out as follows. Thin films of CdS and PANI-CSA consist of glass, with a surface pressure of 12 mm N/1 and a compression rate of 20 mm/min at room temperature. After several runs, the compression rate is increased to achieve stability in the multi-layer deposition. The next precipitate is dried after 30 min, and the substrate is dried without drying. Subsequently, 25-layer, 21-layer, 19-layer, 15-layer, 9-layer, and 3-layer LB films are deposited on the glass layer [100].

#### 4.1.5. Layer by Layer (LbL) Self-Assembly Method

A thin film of PANI is deposited on the glass layer by layer with the self-assembly method. The bottom layer of the glass is first cleaned with the wafer: RCA-1 silicon [101]. In this method, about 325 mL of deionized water in the glass is added with 65 mL of ammonium hydroxide and then incubated at 70 °C–75 °C. Then, 65 hydrogen peroxide is added and heated; a silicon wafer is then added to the solution for 15 min. After washing several times, the silicon wafer is run under water. As a result, the water surface has only one organic layer that deposits on the surface of the wafer. After cleaning, the glass is immersed in the PANI solution with the addition of some rubber particles of adsorption. Once depositing the first layers, the surface is washed with distilled water and is dried out in N2 gas. Finally, the next layer is deposited similarly. Many layers (10 layers) could be coated on the glass layer by using this method [102].

#### 4.1.6. Spin Coating Method

This technique is widely used for the deposition of films of organic semiconductors with any glass. Some polymer solution will be injected onto a semiconductor wafer, which spins at the chosen speed. When the solution runs out radially, the thickness of the fluid layer will be reduced. The vaporization of solution leads to the homogeneity of the film. The first step involves distributing some solutions on the glass. The glass surface can be pre-filtered with a connector to improve wetting [103].

#### 4.1.7. Drop Coating Method

In this technique, the sample dissolves in a suitable solvent that can be stirred by a magnetic stirrer. The solution is filtered and washed with deionized water. The final solution can be used for coating. A clean bed is placed on a flat surface while the solution is placed on the bed by dropping. Hence, the coated bed will be dried in a vacuum for 4 h. To obtain a thicker layer, the second layer is prepared the same way as the first layer. As a result, the glasses are removed from the vacuum and ready to use as a thin film [104].

#### 4.1.8. Nanopatterning Method

In this method, with photolithography, the surface is first covered by light-sensitive light and exposed to ultraviolet light from side to side with a mask. Both light resistance (positive resistance) or exposed areas (negative resistance) can be washed to obtain a positive or negative image on the surface of the mask [103].

#### 4.1.9. Inkjet Printing Method

This method is mostly used to make films of an organic semiconductor. It is well-matched with dissimilar layers and simple to carry out. The possibility of creating a non-mask and non-contact pattern is prepared at low ambient temperature. Here, a computer inkjet printer with simple modifications can be used to create a pattern of processable nanoparticles on various layers such as semiconductor materials, glass, opaque, and paper.

#### 4.1.10. Screen Printing Method

This method is used to produce interconnectors between electronic components in the circuit board. It is applicable to materials with high viscosity, such as conductive materials, dielectric adhesives, UV-resistant materials, and several adhesives. It is a simple and environmentally friendly printing process. Here, a razor blade passes through screen paths and impulses via a screen. It is then spotted with a template to produce open parts, resulting in a conductive layer for the circuit panel [105].

#### 4.1.11. Line Patterning Method

This technique is the simplest and inexpensive process provided by Mac Diarmid et al. [106]. It is used to construct filter resistors. The line patterning method is as follows: (i) draw a negative image for the pattern requisite with PC project software; (ii) deposit a conductive polymer on the glass; (ii) eradicate the printed mask with glass acoustics in toluene.

#### 4.1.12. Nucleation Method

In general, particles precipitate at specific locations in the early stages, leading to the accumulation of particles in identified nuclei or clusters. Nuclei can expand transversely, forming a layer in a small thick area as new particles. Increasing the size of nuclei is complemented by new deposit particles at newer locations of undiscovered parts of the glass surface. The new nuclei rise laterally as the elevation with the arrival of particles. The gap amongst cores gradually decreases, and a continuous film with a medium film thickness is determined eventually. This theory involves the formation of the film, often on an external glass surface, which plays a significant role in nucleation. It is recognized as a heterogeneous nucleus. The nucleation process will be called homogeneous if materials similar to the vapor atom comprise the substrate. In this case of the formation of nuclei and clusters from the vapor stage, the bed surface must be smooth, empty, and free of defects. The general process of adding, absorbing, and disposing of migrating particles is called nucleation. The materialization of a thin film and its derivatives includes phase transfer, adsorption by cluster formation, and nucleation. Several theories are used to illuminate the nucleus and cluster materialization [107].

### 4.2. Physical Methods

The physical technique shows the preparation and characterization of thin films of PANI at ambient temperature by electrodeposition methods. Various introductory factors, such as dopant type, deposition potential, monomer alloy, and declaration period for a well-developed and uniform PANI thin film, are illustrated in different ways. The electrical deployment strategy consists of combining thin films of damaged types by altering their oxidation state with electricity. Therefore, the final position has a variety of crucial points concerning the others that work with the techniques used to advertise thin films [108]:

1. This is an inexpensive way to make thin films.
2. Sediment must be selected in a particular area, and the thickness of the deposit will be fully controlled by trying the load.
3. This is an extremely useful method of manufacturing thin multilayer materials.
4. The deposition will be performed at low temperatures.
5. The deposition can be performed in many ways.
6. The process will be prepared much easier than competing methods.
7. Different types of morphology and compounds will be obtained for polymers, mixtures, and different mixtures (Table 1).

#### 4.2.1. Galvanostatic Method

**Figure 17:**
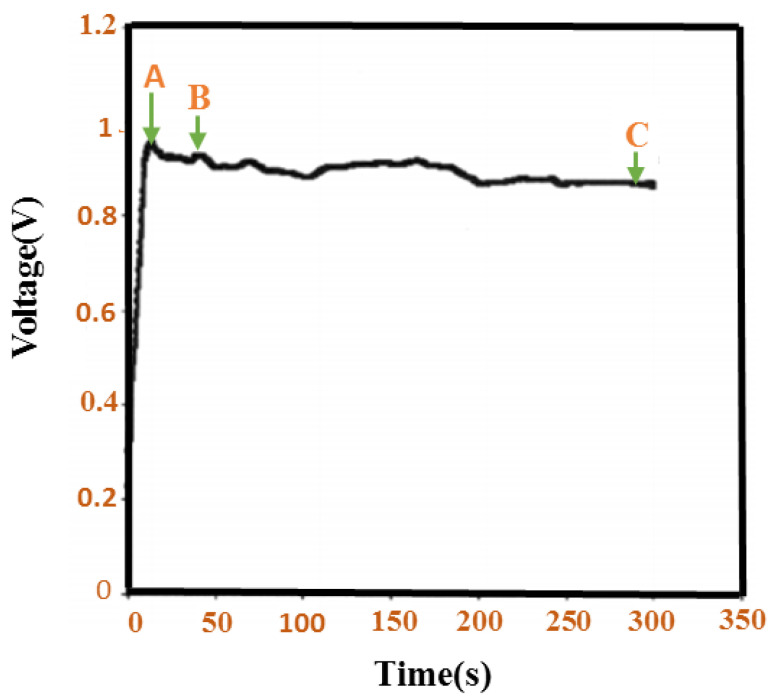
PANI film with galvanostatic deposition. Reproduced from Eur. Polym. J., 125, Kumari Jangid, N.; Jadoun, S.; Kaur, N., A review on high-throughput synthesis, deposition of thin films and properties of polyaniline, Copyright (2020), with permission from Elsevier.

Figure 17 illustrates the temporary approval of the potential and the time of the PANI film at a constant flow thickness. The possibility of kernel design and PANI enhancement is selected. Initially, given the creation of a space charge zone, this potential increases to + 0.92 volts. In B, aniline oxidation occurs on the substrate, and a permanent improvement of the PANI is observed during the year AC. Films that are predictable under current conditions are thinner and have less support for refined steel layers. In this sense, the PANI filmmaking method is completed. In B, aniline oxidation occurs on the substrate; during the year AC, an invincible improvement in PANI is observed. The thin films available under existing situations are reliable, thin, and likely to form layers of refined steel. Subsequently, this production strategy of PANI films has been suspended.

#### 4.2.2. Potentiostatic Method

**Figure 18:**
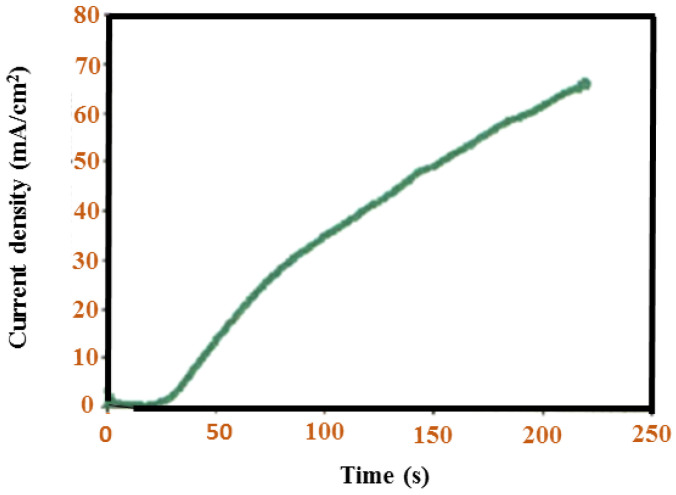
PANI film with potentiostatic deposition. Reproduced from Eur. Polym. J., 125, Kumari Jangid, N.; Jadoun, S.; Kaur, N., A review on high-throughput synthesis, deposition of thin films, and properties of polyaniline, Copyright (2020), with permission from Elsevier.

A latent decrease in current can be observed because of the adsorption on the electrode surface of the particle. Continuous increasing flow after initial collapse indicates a parallel PANI nucleus approaching the autocatalytic recovery of PANI. A transient existing in the precipitation of potentiostatic acetate is the result of using H_2_SO_4_ as an electrolyte. The color is light blue because of the H_2_SO_4_ electrolyte utilized at the assembly of 0.45 mL aniline; a current transient follows nature, as demonstrated in Figure 18. The electrical oxidation of aniline produces a powder that sticks to the electrode at a constant potential. Thus, it is not used to film precipitate PANI in subsequent work.

#### 4.2.3. Potentiodynamic Method

**Figure 19:**
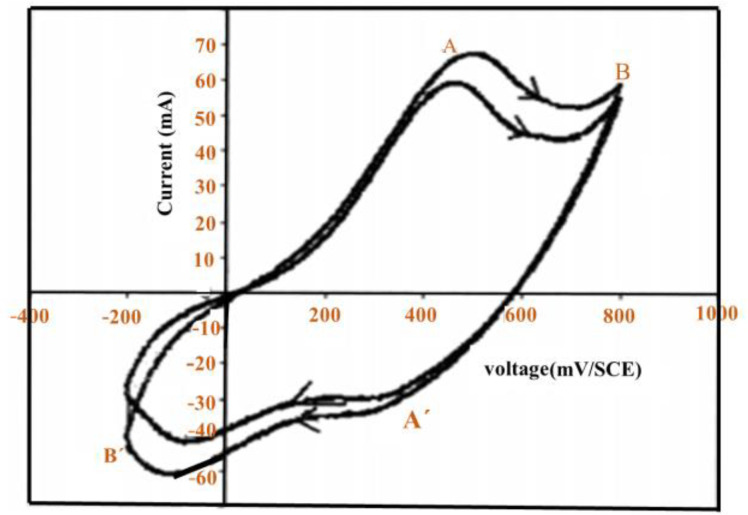
PANI film with potentiodynamic deposition. Reproduced from Eur. Polym. J., 125, Kumari Jangid, N.; Jadoun, S.; Kaur, N., A review on high-throughput synthesis, deposition of thin films, and properties of polyaniline, Copyright (2020), with permission from Elsevier.

Figure 19 illustrates the cyclic voltammogram (CV) diagrams of PANI deposition with a bandwidth of 50 mV/s in the potential range of between −0.2 V to + 0.8 V/SCE under the potentiodynamic mode. Decomposing the peaks, A (+0.45 V/SCE) and analysis for aniline oxidation occur within the potential region of the AB port (up to 0.8 + V/SCE) PANI progression. When the cycle considers −0.2 V/SCE, PANI is completely reduced in the leucoemeraldine (LE), and a yellow thin film is obtained. With the first oxidation at A (0.45 V/SCE), the reduced state does not completely oxidize, resulting in the emerald state (EM). The film becomes green. At point B (0.8 V/SCE), the EM changes into complete oxidation to produce PANI, and a blue-violet thin film is obtained. In this method, which is used for PANI storage, the cathode is tested using ten rotations under 20.2 and +0.8 V/SCE in 50 mV/s. Oxidation of aniline does not occur during 20.2 to +0.45 V/SCE, whereas PANI oxidation and progression occur in +0.45 to +0.8 V/SCE. Deposition films of PANI survive in three distinct periods. PANI films that survive potentiodynamically are subjected to further investigation and production of heterogeneous connections.

#### 4.2.4. Thermal Evaporation 

This technique is used for the deposition of alloys, metals, and various composites; it involves the evaporation of materials under a vacuum with thermal energy. Thermal evaporation allows the vapor flow of the material to reduce and turn into a single layer to the adhesive film of the chosen thickness. The characteristics and quality of the layer depend on the amount of evaporation, bed temperature, and pressure. The uniformity of the layers is influenced by numerous factors, including the geometry for evaporation and their space from every substrate [107]. This method is passed in various ways, such as resistive heating, in which materials are generally heated by resistive filaments of refractory metals with or without ceramic liners, such as Nb and Ta. Other cases include flash evaporation, wire blast method, laser evaporation, electron beam evaporation, and RF heating. To overcome the disadvantages of the resistance method, an efficient heat source can be used with an electronic material bombardment that uses the evaporation of all material at rates from angstrom to micron per second. As a result, chemical compounds and their structures can be changed by this method due to extreme heat or the bombardment of high-energy electrons [109].

#### 4.2.5. Sputtering Method

Expulsion for atoms that started the surface for a substance with energetic particles as scattering will be called sputtering if the repulsion is positive because of ion bombardment. The extracted or scattered atoms will be compacted on the glass to shape a thin layer. It will be called glow discharge spray if ions will be completed with discharge phenomena due to the presence of an electrical field among double electrodes in the low-pressure gas. Parameters that affect the performance of this method are pressure gas, sediment distribution, values of current and voltage, and cathode size. Sedimentation/evaporation rate remains constant as long as the current–voltage and density will be achieved with a tuned power source with automatic pressure control. The spraying process will be classified as RF spraying, sensitive spraying, triode spraying, ion spraying, ion beam spraying, and magnetron spraying [110].

## 5. PANI Features

The unique properties of nanoparticles are introduced by a conductive polymer. In any given study, it is clear which PANI is used for making a nanocomposite. Mineral metal oxide nanoparticles are often incorporated by conductive polymers to complete the chosen properties. Recently, PANI inorganic nanocomposites have shown interest because of several exciting physical properties and potential applications. Maeda and Armes approved chains of pioneering that work in nanocomposites of conductive polymers [111]. SiO_2_, SnO_2,_ and so on, combined with PANI can obtain a stable colloid dispersion [112,113,114], which is applicable to biomedical fields. Nano-manganese(IV) oxide is obtained by PANI for the production of water-soluble nanocomposites [115]. WO_3_ [116] plus Prussian blue [117] is produced by electrochromic properties. Some scientists have developed nanocomposites using Cu_2_O nanoparticles in PANI to improve optical, mechanical, and electrical features [118]. Kondawar [119] prepared nanocomposites by combining ZnO in the PANI matrix and found an increase in their electrical conductivity in the nanocomposites. He also found different conductivity of nanocomposites, which is related to a variation in the polymer’s doping state [120]. Currently, numerous researchers have described that metal oxide/polymer nanocomposites and their combination, embedding, or mixing metallic oxide in polymers that increased the thermal stability and optical features, such as absorption, conductivity, and oxidization resistance for metallic oxide [121].

**Figure 20:**
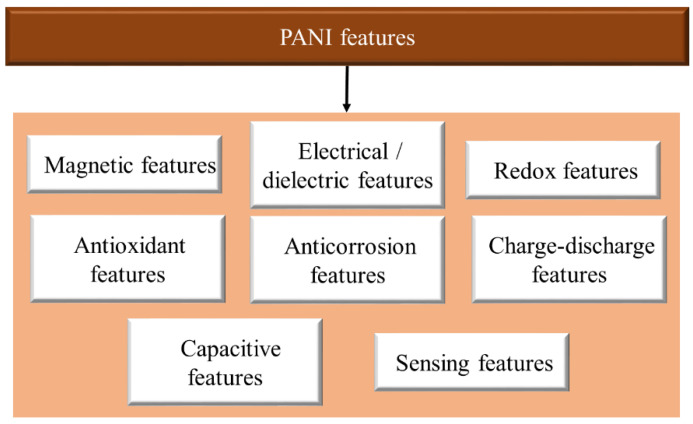
Features of PANI.

Another study has been conducted on the Cu_2_O/PANI compound to explain the proposed photocatalytic mechanism underlying the enhancement in photocatalytic activity, in which photocatalysts work by increasing the stability and photocatalytic activity of the compound. Therefore, PANI is related to various properties, including magnetic, electrical, and dielectric, redox, antioxidant, and anti-corrosion, charge–discharge, capacitive, and sensor properties (Figure 20).

### 5.1. Magnetic Features

PANI pays attention to magnetic behavior due to its high rotation density [122]. These properties of CP have been extensively considered because they provide information on unpaired rotations and carrier types [123]. The paramagnetic nature is closely related to the electrical and magnetic properties of the PANI conductor due to HCl doping. Lu [124] prepared PANI-Fe_3_O_4_ composite nanotubes, which show highly magnetic behavior via the ultrasonic radioactivity method and their magnetic features. Similarly, PANI-Fe_3_O_4_ composite nanorods are prepared by the self-assembly method. In comparing methods, the samples obtained by ultrasonic irradiation distribute Fe_3_O_4_ particles. These properties have been investigated in 1D-CPs, such as Fe, Ni, and Co nanocomposites [24]. Given that polarization is affected by H bonding in methanol and the PANI structure, a significant ferroelectric response has been observed in PANI nanotubes. Djurado [125] researched PANI film with the di- (2-butoxy ethoxy ethyl) protonic acid 4-sulphofthalic acid and found that it does not exhibit magnetic behavior with zero magnetism. The magnetic manners of PANI have also been investigated in the existence of chloranil during electrical oxidation [126]. PANI rotational sensitivity indicates that the term is used for paramagnetic centers, which exist in charge holders that are primarily implanted in metal fields, as well as in the backbone of amorphous polymers. In this rotation, the electron communicates with the samples and exhibits anti-ferromagnetic features such as paramagnetic sensitivity [127].

Huang [128] prepared composite films of PTS, HCl, and CSA SrPr_0.2_Fe_11.8_O_19_/PANI and films of HCl-PANI via the sol–gel technique and oxidative polymerization in situ. He observed that these composite films do not fluctuate in terms of magnetic properties. Electromagnetic losses lead to four types of films, ranked from strong to weak as follows: HCl-PANI film, SrPr_0.2_Fe_11.8_O_19_/(HCl-PANI) composite film, SrPr_0.2_Fe_11.8_O_19_film, and SrPr_0.2_Fe_11.8_O_19_/(CSA film). As a result, PANI material is greatly reduced due to the loss of its electromagnetic features in composite layers.

### 5.2. Electrical/Dielectric Features

PANI is highly attractive amongst polymers due to its thermal properties and environmental stability. The source of the normal base and metallic phase of PANI is unknown [129]. Polyacetylene is the first electrical conductive polymer used as a doped organic polymer, which was discovered in 1977 [130]. The virgin polymers are converted to a metallic organization during the doping method. The electrical conductivities for polymers are improved by growing the doping level, whereas PANI conductivity is improved by several orders via doping. These features of polymers plus their nanocomposites can be assumed on the description for several models developed with experts.

Numerous reports have focused on the environment for carriers in the highly doped metal state. However, under a large concentration of electron conductivity on the Fermi surface in the extremely doped state, the carriers are localized, where they do not participate in transport except by jumping. Given that conduction is carried out via local electrons, electrons jump from one site to another through an energy barrier. When two particles are divided with a potential barrier, the carrier will be moved to begin from one cross to another cross through another tunnel barrier. The procedure of moving carriers beginning from one cross to another, similar to jumping in a structural disturbance, is the main source of localization in polymers. Charge transfer is an essential phenomenon that results from the connection between polymer temperature and AC conductivity in a polymer system. Conductive polymers are generally either polycrystalline or amorphous and have a significant degree of disruption. The existence of minor crystallization and impurities can disrupt a polymer.

Conductivity is influenced by numerous defects in the polymer. Cargo is transported between local sites by telephone jumping due to local cargo carriers instead of runway transmission. The activation energy involves each hop, and the jump occurs near Fermi energy, which is predominant in low temperatures. The hop acquired prevails near the maximum state density at high temperatures. In general, an electron jumps farther away from the nearby neighbor to decrease the energy required for the hop. Crystallization and irregularity of polymers are investigated by XRD spectroscopy. The filamentary environment of several conductive polymers can cause localization by decreasing the actual dimensions for the electrons housed in the polymer chain package [131].

In a complete crystal through periodic potentials, the electron wave utilities form the local block waves [132]. Network and impurity defects in disorderly systems are presented backward through sprinkling. Anderson (1958) investigated this phenomenon concerning the effect of localization and transfer of metal insulation due to irregularities. The electronic structure of the structure is intensely related to the degree of disturbance. The vibration of energy at accidental potentials expands bandwidth and makes smooth bands. However, the main bandgap amongst the conductor and the capacitance bands for a semiconductor can be closed due to the presence of these band extensions. Mott deliberated the consequences and the limited mass of states NEF, which was made at the Fermi level (EF) among the kinesis edges in 1979. After the 1940s, it became clear that the transistor result of semiconductor materials, such as the doped semiconductor, cracked the apparatus at low temperature behavior, which Hong and Gliessman had established as the previous kind of performance in the overall conductor.

In 1956, a novel conduction procedure was proposed by Mott, who reported that charge carriers use heat-activated tunnels to transfer electrical current from an employed site to an empty site. A large number of transportation theories were developed according to the Miller and Agrahan model in the 1960s; even if the theories are more widely accepted, the models have little difference amongst one another. A more complex treatment method (called leakage theory) has been proposed, and it is still the most reliable in terms of evaluating the electrical coefficient as the thermal power and thermal conductivity of non-crystalline semiconductors; it is also considered for a one-dimensional metal chain. Localization of charge carriers occurs even for frail perturbations due to quantum interference with unstable static returns. [133].

Reciprocally, severe irregularities are required in 3D systems for localization. The effects of localization on conductive polymers in disordered (somewhat crystalline) homogeneity may stem from one-dimensional localization in chaotic areas. Regarding the conductivity of polymers such as fillers, the degree of crosslinking among polymer chains similarly disturbs the electrical properties of these composites. [134]. In general, the seepage principle is applicable to illuminate the dielectric properties and the electrical conductivity of conductive polymer composites; nevertheless, it does not observe the low penetration threshold values perceived in numerous conductive polymer composite systems. Wesling proposed the theory of imbalance for conductive polymer composites in 1998. This theory, which accounts for the surface interfaces amongst the host matrix and conductive polymers, can explain low penetration threshold explanations. Conductive polymers are significant applicants in electromagnetic and electronic applications. Therefore, PANI has remarkable conductivity plus capability, and the dielectric features are simply adjusted in combination with mineral nanoparticles. When considered as nanocomposites, their electrical and dielectric properties vary from the main material.

Several groups have described the electrical conductivity and dielectric properties of various conductive polymer compounds [135]. The conductivity for these methods is influenced by various parameters, such as concentration for conductive fillers, size, shape, orientation, and surface contact between the host matrix and the filler molecules [136]. The nano form of plaster particles controls the ability to form a conductive network that leads to a large growth in conductivity. Furthermore, the interaction of the physical features for the matrix and the capsule/matrix particles affects the aggregation for the encapsulation stage, which then disturbs the dielectric features of the nanocomposites. The crosslinking degree amongst polymer chains disrupts the electrical features for the composites in the case of conductive polymers. However, this theory is usually utilized for the electrical conductivity and dielectric features of conductive polymer composites [134]. The low penetration threshold values observed in many composite systems do not explain conductive polymers. Wesling suggested a non-equilibrium principle for conductive polymer composites that explains the low diffusion threshold observations while justifying the surface interfaces between the host matrix and conductive polymers.

The composition of PMMA enhances the mechanical and optical features of PANI film, which films show high conductivity, low adhesion, low inherent stress, and low refractive index [137]. Thin films of PANI/PMMA compound synthesized under vacuum evaporation employ short-chain oligomers. The optical bandgap increases due to the PMMA concentration, thereby completing the formation of a homogeneous composite. This optical transmission of virgin films increases with increasing PMMA concentration from 70% to 85%, whereas the refractive index decreases from 1.822 to 1.571. After adhesion rises from 6.86 × 10 ^4^ to 12.26 × 10^4^ N/m^2^, stress decreases from 16.98 × 10^8^ to 10.50 × 10^8^ N/m ^2^. High conductivity p-type semiconductors of PANI and PANI film include camphor sulfonic acid (CSA)-doped PANI thin films with a reported CSA weight ratio of 1: 8 [65].

### 5.3. Redox Features

PANI exhibits various shapes that are reversibly converted by accepting or removing electrons. The decrease in the polymeraldin form and the base form of the polyalcoamerldine changes in a positive direction with increasing scan rate [138]. Gospodinova et al. [139] examined HCOO^−^, which is similar to extremely hydrated anions and demonstrated the propagation of PANI diffusion in structured thin films in an acidic medium. The electrochemical action of PANI film is arranged by PANI casting of N-methyl pyrrolidone solution (PANI-NMP) and additional film composed of a mixture of dichloroacetic acid and dodecyl benzene sulfonic acid (PANI-DBSADCA) by dissolving PANI. A comparative study of both was conducted with aqueous NaCl solution (or HCL) as an electrolyte. Finally, results indicated that PANI-NMP and PANI DBSA-DCA films were active up to pH < 5 and at pH < 2, respectively, while PANI DBSADCA weight increased with decreasing Na^+^ movement and weight. The film of PANI-NMP is enhanced with oxidation due to Cl motion. Thus, PANI is applicable in redox-active nanojunctions [140]. Scanning electrochemical microscopy (SEM) is utilized to prepare nano-functions that show conductivity in the range of 10^−7^ and 10^−8^ S. This current can be controlled when it is less than 10 oligoaniline chains because the smaller number of chains will be able to connect two electrodes then connections will be very stable in the environment.

### 5.4. Antioxidant Features

The 2,2-Azino-bis (3-ethylbenzothiazoline-6-sulfonic acid) diammonium salt (ABTS) is prepared to determine the antioxidant properties of PANI powder [141]. The reduced procedures of PANI illustrate the maximum radical scavenging properties matched to their somewhat oxidized procedure. The radical scavenging features (DPPH %) of PANIs are not reliant on the conductivity and surface of PANI but dependent on the dissimilar oxidation states for PANI. So, ABTS shows that the antioxidant feature has been improved by reducing the PANI oxidation level. When dried at temperatures above 200 °C, PANI shows a quick decrease in free-radical scavenging properties [142].

### 5.5. Anti-Corrosion Features

Even though PANI is considered an essential material for oxidization mechanism coverings, it can also be used in protecting against corrosion, which was first reported in the 1980s. PANI was utilized to effectively protect oxidization in stainless steel alongside proton interchange membrane fuel cells. Recent research established which PANI sulfate coats are more suitable than PO_4_^−3^ polyaniline coats to prevent stainless steel from erosion [143]. Likewise, PANI sultanate coats afford anode avoidance for stainless steel substrates. The significance of PANI tungstate for protecting steel against erosion is also proven [144]. The time potential curves achieved by polymerization electrochemical galvanostatic for aniline with low carbon steel in (COOH) _2_ solutions indicate two divergent steps: formation of polycrystalline iron oxalate supplement and solubility of the carbon steel surface and electrochemical polymerization of aniline. Electrically synthesized PANI coatings are also used to protect mild nickel-plated steel against corrosion [145]. Polarization curves show an insignificant increase in the erosion potential of PANI aluminum composite (6061-T6), which is related to empty metal. Hence, insufficient shelter is acquired with a PANI undercoat [146].

Alternatively, the reduction of Al destruction due to galvanic oxidization for copper- PANI aluminum galvanic pairs in the marine ambient has been obtained by Vera et al. because of the presence of PANI in the copper aluminum structure, which is due to the reduction of the oxygen reduction reaction (ORR). Electrochemical impedance spectroscopy (EIS) demonstrates the coating fixation of Al compounds (AA 7075, AA 2024) by PANI that was synthesized in solution (COOH)_2_, then it displays a resistance higher than 10^6^ Ω cm^2^, while composites coated by epoxy resin simply, in this case, the value of resistance can be fewer than 10^4^ Ω cm^2^ [147]. It is observed that the erosion resistance of PANI film for aluminum composite (AA 7075) has been increased from 70% to 90% by usage in Ce salt solution. Differentiations of three sorts for PANI/metal edges are performed by the Kelvin probe that matched with the regular acquaintance of PANI-protected metal to wet and dry air and wet N2 [148].

### 5.6. Charge–Discharge Features

LiPF_6_ doped electro-sprayed of PANI on the top of aluminum foil has been utilized as cathode material for Li batteries at ambient temperature via the electrospinning method [149]. Hence, the cells deliver an extraordinary discharge capability and approach the maximum theoretical capacity, which decreases to 61% after the accomplishment of 50 cycles. Likewise, PANI polysulfide is utilized, for example, in a cathode with the substantial elevation energy of Li/S batteries [150]. Recently, researchers focused on rechargeable batteries related to PANI compound materials.

### 5.7. Capacitive Features

Electrodes and PANI supercapacitor devices involve the boundary between carbon-based supercapacitors; these devices are also complicated intermediate energy units. PANI is applicable in capacitive applications due to a rapid redox reaction in the bulk of PANI. It gives a capacitive response and illustrates superior specific energies compared with two-layer capacitors. Moreover, it is obvious which PANI is more conductive in the environment and has a stronger influence than inorganic battery tools. However, the lifespan for large capacitors that are pure PANI, such as dual-layer capacitors, is poorly compatible with the material for PANI and experiences contractions in the charge and discharge cycle. Consequently, it is observed that sufficient evolution is made in the synthesis of compounds of PANI materials, for example, PANI—carbon nanotubes (PANI-CNT), PANI—graphene, and so on by exceptional capacitive properties [151].

Kim [152] prepared PANI films on ITO flexible substrates (polyethylene terephthalate-indium tin oxide) using the casting method by changing the PANI value in the range of 0.04–0.16 g. Morphological study illustrates a porous vermicular form, whereas an electrochemical supercapacitor in a 0.5 M LiClO_4_ + PC electrolyte was investigated. PANI thin films by 0.08 g of PANI illustrate relatively complex existing density, specific capacity, and storage capacity than PANI films. They established a 0.08 g droplet. Thin films of PANI can be utilized as capacitive cloud electrodes to increase energy density, stability, and strength. The influence for PANI value similarly shows its exceptional features for using a supercapacitor.

### 5.8. Sensing Features

Thin films of PANI are applicable for use in sensors [153]. A simple and convenient method for measuring very small amounts of mercury, water tissues, and CH_3_-Hg in fish is developed through a micro-column packed with PANI. A glass carbon electrode is made with the comfort of a PANI coat and is used to detect Cd^2+^ and Pb^2+^ in CH_3_COO by buffer solutions [154]. The modified PANI Langmuir–Blodgett PANI film of carbon electrode was used to detect Ag^+^ ions in a voltammetry sensor to determine Ag^+^ ions [155].

PANI thin films are only utilized in sensors due to their fast response time and extraordinary surface area. They are utilized in the finding of numerous gases, such as CO, ammonia, and LPG. For gas detection steps, thin films of PANI are placed in the gas chamber vertically, and the practical modifications are measured. Hence, the adsorption of a gas on the thin layer increases when cleaning the gas in the chamber, and this shows the feedback between the gas particles and PANI. A simple and inexpensive method of potentiodynamic sediment electrode is used by Mello [156] to determine the value of PANI thin films. The researcher observed that PANI films of electrical deposition are highly sensitive, linear and stable, and the films can be a competitor to commercial pH and biosensors.

Thin films of PANI were fabricated using a spinner and examined by exposure to a damp sensor by Kumar and Yadav [157]. Thin films of PANI-TiO_2_ using an in situ self-assembly technique have an upper reaction, good reversibility, and quicker reaction recovery speed than those based on PANI-TiO_2_, which similarly have detectable reproducibility, selectivity, stability, and low concentration is (1 ppm) to NH_3_ [158]. They also suggested a model of sensing device development sensing features. They observed that the ternary film of the sensor could be an effective approach to developing better-performing ammonia sensors at room temperature. More recently, the team explored the effect of CeO_2_ nanoparticles on the performance of PANI-based NH_3_ sensors at ambient temperature [159]. The sensors exhibited enhanced reaction, reduced recovery of time, linearity of full concentration response, great reproducibility, very good selection, extraordinary longstanding stability, excellent flexibility, and very low obvious concentration.

Khanikar and Singh [160] established an essential fiber optic pH sensor covered by a PANI polyvinyl alcohol (PVA) compound layer. This sensor is made by replacing polymer fiber with a coating of a pH-sensitive PANI-PVA layer and shows extraordinary sensitivity effects for 2.79 μW/pH for pH 2–9. Moreover, they found that ionic strength and ambient temperature affect the performance of the sensor. Finally, research showed that the planned sensor has very good durability and stability [93]. Hence, PANI is embedded in the vanadium pentoxide (V_2_O_5_) matrix, and it monitors the performance of the ammonia sensor.

## 6. PANI Applications

**Figure 21:**
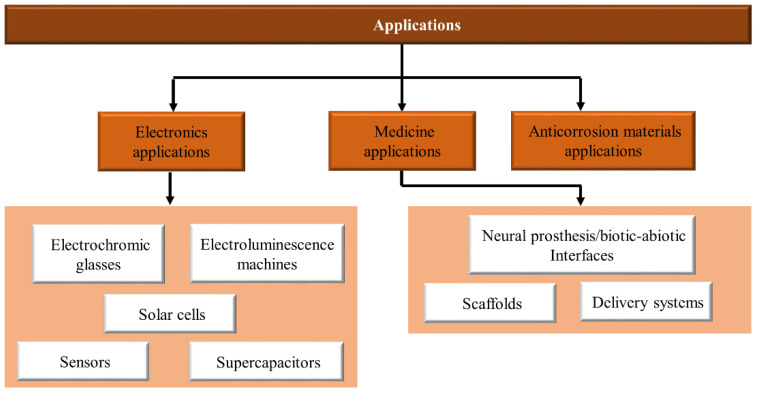
Application of PANI.

Conductive polymers have unique properties due to their inherent electrical activity, and their diverse applications have led to a variety of applications. PANI is an extremely conductive polymer; given its easy synthesis, unique properties, and low cost, it is attracting remarkable consideration in various applications (Figure 21).

For electrochromic glass, discoloration depends on the electric current of the chains. PANI electro-luminescence structures are used in the construction of LEDs. Solar cells have also been promoted by PANI in the commercial use of low-expense solar cell fabrication technology. PAN-based solar cells reduce invention costs and increase energy efficiency. Finding materials in different applications is a requirement. Hence, PANI-based sensors are built for diagnostic purposes, including gas and glucose sensors. Given the growing development in industrial aspects and energy consumption technology, energy storage devices, such as supercapacitors that highlight optimal consumption, have been produced. PANI with extraordinary conductivity, various redox stresses, and low cost is used in cloud capacitor development. Currently, medicine is about engineering, and advances in this area require new intellectual technologies. Neuroscientists require devices that compensate for nerve weakness and contribute to the development of neuroscience. Scaffolding has been utilized for correcting organ disorders, and biocompatibility conductive scaffolding has good bio-counterfeit properties. In addition, PANI applications have received much attention in delivery systems; as a result, original delivery structures such as electro-drug delivery systems are being explored. The application of PANI as an anti-corrosion barrier has yielded successful results [161]. In this review, the application of PANI is categorized as shown in Table 2.

### 6.1. Electronic Applications

#### 6.1.1. Electrochromic Glasses

**Figure 22:**
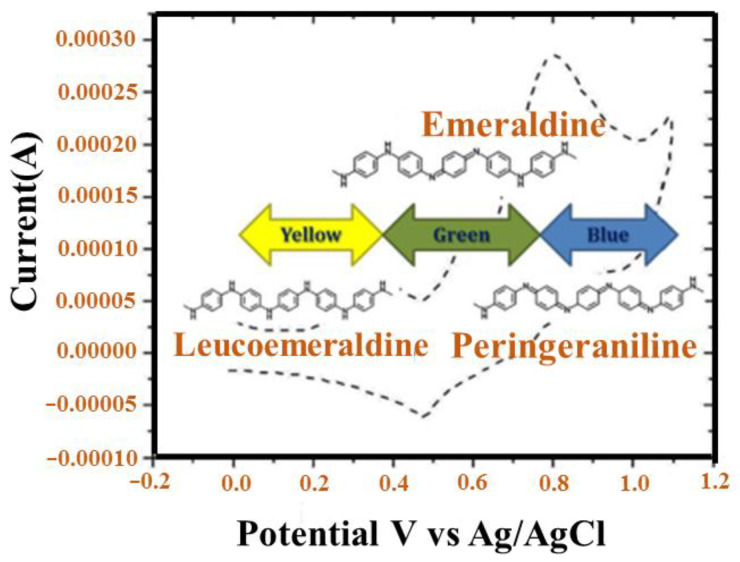
PANI in place of smart glass with oxidation/reduction states. Reprinted from Fundamentals and Emerging Applications of Polyaniline, Zarrintaj, P.; Vahabi, H.; Saeb, M.R.; Mozafari, M, Application of polyaniline and its derivatives, pp. 259–272., Copyright (2019), with permission from Elsevier.

The smart or electrochromic glass will change color if an electric current pass through it. The degree of turbidity of the glass will be determined by the grade of voltage transfer over an electrochromic glass. The oxidation/reduction state of intelligent material can be detected even with the naked eye. PANI, which is capable of reflecting blue, such as a crystal medium in the passageway for electric current, is amongst these materials. The opacity for glass is altered in color, translucence, and modes [162]. This characteristic leads to the development of technologies with internal and external applications. For example, the darkness of a car window can be adjusted by an electric current or by using current over a wide range of potentials; for example, antiques in an exhibition hall in a museum are guarded against UV rays or synthetic light (Figure 22) [161]. Here, enormous smart glass is established related to the reflection appliance that acts as a mirror [163]. To enhance its smart features, PANI must be covered by tin (IV), oxide (SnO_2_), or indium tin oxide (ITO) glasses by forming a film. The thickness of the thin film can be organized by polymerization, such as electropolymerization. You can also change the color of the glass by changing electrical energy from green to yellow or extra crystal clear. A fast response time is to be expected, in moments or fractions of a second, followed by a long lifecycle [167]. A reversible color change time in the potential sweep can be proportional to the ion dispersion coefficient [168]. PANI exhibits several colors that are related to its oxidation state and its electrical current effect-like states [169].

Consequently, the color depends on variations in the oxidation state. In addition, the sort of dopant and electrolyte used can reduce the oxidation state separately or simultaneously. For example, non-precious solvents show a peak of lone redox. The use of a certain solvent can result in some peaks that are determined by their electrochromic properties [170,171]. The composite of PANI for organic/mineral materials can be a practical method to perfect the color of electrochemical utilization, such as for smart windows and displays [172]. PANI/Clay presents complex flow density because of the proper interface between the clay lamellae and PANI, which leads to an increase in the conjugate composition [173]. PANI-derived graphene oxide dye yields are high, but colorful electrochromism is obtained at different potentials [174]. A suitable PANI layer needs a strong connection between the substrate and PANI. Hence, alteration in PANI with ITO through its deposition by 4-amino benzyl phosphonic acid was investigated and resulted in increased stability and electrochemical activity of PANI [168]. Hydrothermal conditions were utilized to synthesize PANI with FTO to reach homogenous PANI fibers in a short period [164]. It was similarly synthesized by polymerization. A uniform cover with a rotating coating was obtained, showing a large diffusion coefficient and a small load transfer resistance at a similar time [165]. PANI-WO_3_ shows a more stable and faster change than PANI [166]. In practice, it is assumed that PANI will be altered with various precursors to expand its scope.

#### 6.1.2. Solar Cells

**Figure 23:**
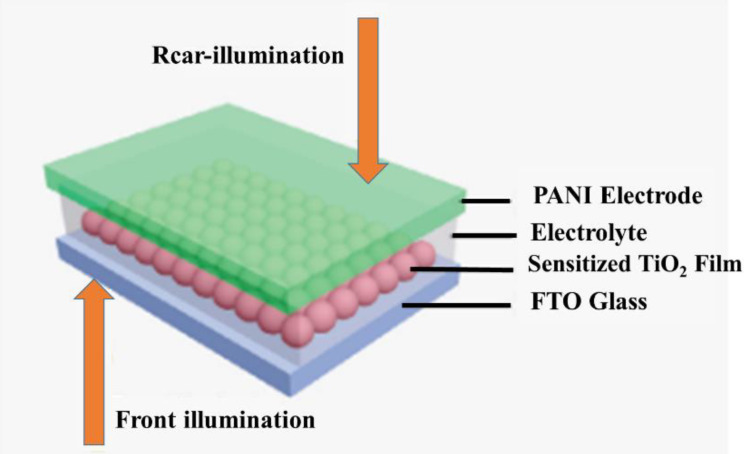
Schematic of the bifacial DSSC related to transparent PANI counter electrode. Reprinted with permission from Tai, Q.; Chen, B.; Guo, F.; Xu, S.; Hu, H.; Sebo, B.; Zhao, X.-Z. In situ prepared transparent polyaniline electrode and its application in bifacial dye-sensitized solar cells. ACS Nano 2011, 5, 3795–3799. American Chemical Society.

**Figure 24:**
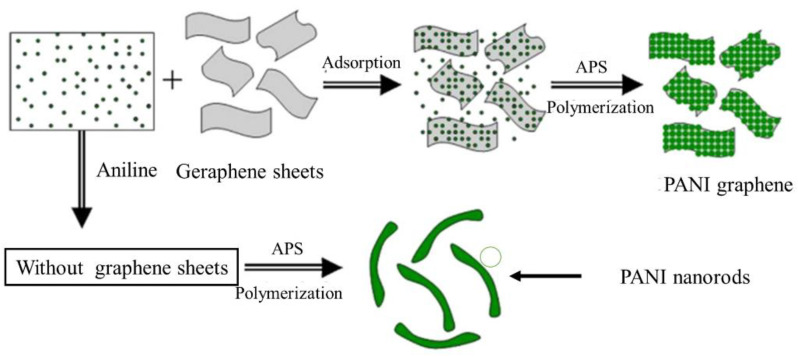
Schematic for the preparation of PANI/graphene hybrid. Reprinted from Electrochim. Acta, 66, Zarrintaj, Wang, G.; Xing, W.; Zhuo, S., The production of polyaniline/graphene hybrids for use as a counter electrode in dye-sensitized solar cells, pp. 151–157., Copyright (2012), with permission from Elsevier.

Dye-sensitive solar cells (DSSCs) are economical, inexpensive, high-performance film solar cells based on an electrolyte and a semiconductor [176,177,178]. In general, they contain a color-sensitive titanium dioxide electrode, a redox electrode, and a counter electrode that is often made of platinum (Pt). The most considerable cost of DSSCs is from Pt, which is changed with carbon-based materials. PANI is utilized in DSSC because of its easy synthesis, low price, and good conductivity. Microporous PANI looks better than Pt electrodes due to complex electrocatalytic rooting activity at I_3_- and a smaller charge transfer ratio of oxidation responses [244]. The absorbency of PANI rises at a particular surface area, progresses the catalytic activity, and increases the efficiency of trapping liquid electrolytes for DSSC [179]. PANI is electrolyzed in FTO glass (Figure 23) by several counteractions (for example SO_4_^2−^, BF_4_^−^, CL^−^, ClO_4_^−^, and p-toluene sulfonate [TsO^−^]) to achieve counter electrodes [183]. Hence, PANI-SO_4_ presents maximum porous medium by a minimum charge transfer resistor and maximum reduction current for I_3_ oxidation feedback [175]. PANI polymerization onto the graphene surface increases the PANI electrical area and overall conductivity (Figure 24) [180]. Therefore, this method improves electrocatalytic activity, such as an anti-electrode in DSSC [180].

**Figure 25:**
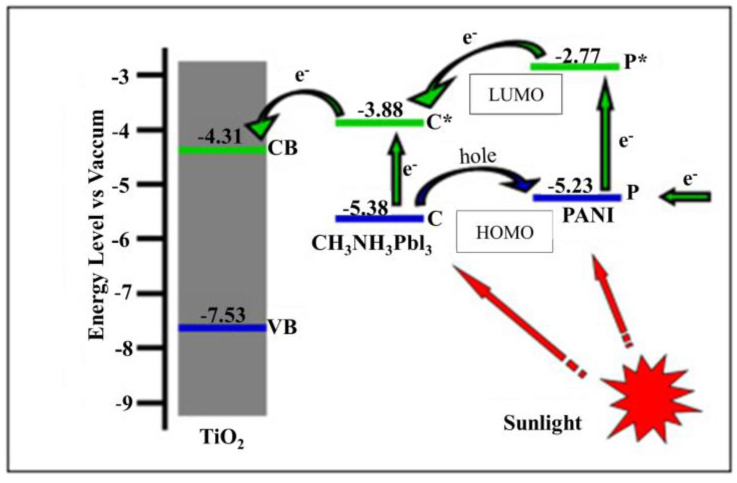
Schematic energy level illustration of TiO2, CH3NH3Pbl3, and PANI. Reproduced from Power Sources, 267, Xiao, Y.; Han, G.; Chang, Y.; Zhou, H.; Li, M.; Li, Y., An all-solid-state perovskite-sensitized solar cell based on the dual function polyaniline as the sensitizer and p-type hole-transporting material, pp. 1–8., Copyright (2014), with permission from Elsevier.

PANI electrical deposition possesses an extraordinary charge transfer apparatus and improved electrical/electrochemical efficiency compared to PANI chemical deposition and electrochemical polypyrrole deposition (PPY) [181]. In perovskite-sensitive solar cells, dual-function PANI plays the role of P-perforating and sensitizing material due to π–π * transmission and ground-based polarization. Depending on the surface, the PANI structure affects light absorption, whereas the porous surface of PANI will be completed by CH_3_NH_3_PbI_3_ and L salt to increase the light absorption distance and carrier mobility [182], as shown in Figure 25. Leveled nanowires of PANI show the high efficiency of solar cells [245]. PANI facilitates the construction of two-way DSSCs at a low cost because of the use of all sides of DSSCs [183].

#### 6.1.3. Electroluminescence Machines

**Figure 26:**
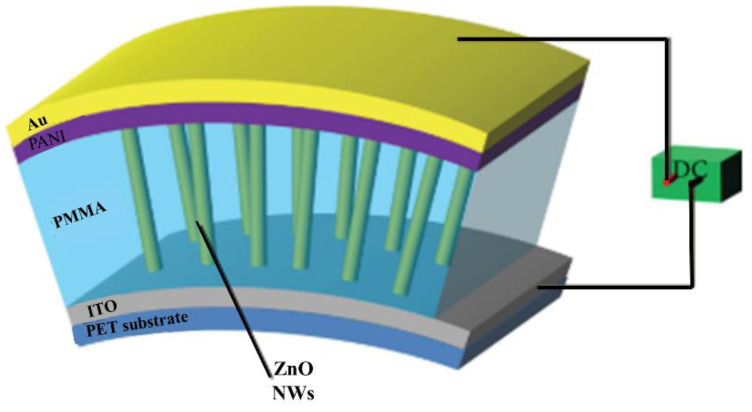
Schematic of the flexible LED device. The simple structure of the device is Au/PANi/ZnO NWs in PMMA matrix/ITO/PET substrate.

Electroluminescence devices can be synthesized by substances that emit light due to the induction of an electric current/electric field. They are a source for light-emitting diodes (LEDs) of a p-n connection diode that may emit rays by applying a suitable voltage. Hence, electrons can be combined with the device’s electron holes to discharge photon energy [184]. A bright color will be obtained with the semiconductor energy bandgap. Inorganic LED (OLED) is a conductive polymer that acts as a perforated injection film. PANI-poly (styrene sulfonate) (PPS) is utilized for increasing OLED performance. PANI-PPS results in more complex performance than commercial PEDOTPPS and leads to increasing hole injection. In comparison, given the high conductivity, medium clarity, and roughness, PANI-PPS shows maximum efficiency and minimum voltage performance [184]. PPS with a conductive polymer, such as its PANI copolymer solution, offers easier doping, solubility, and film quality for thematic monitors/screens than conventional PEDOT-PPS [185]. PANI self-doping based on aniline and (aminobenzene sulfonic acid) is assembled in its ITO glass to create a perforation injection film in two-layer electroluminescence with an orange electroluminescence display compared with a single-layer electroluminescence device [186]. An organic-mineral ray-emitting diode related to ZnO/PANI nanowire (type n/p) is similarly utilized for water diffusion, which is obtained due to the recombination of the electron boundary in the conduction band and the holes in a wide light range (Figure 26) [187].

#### 6.1.4. Sensors

**Figure 27:**
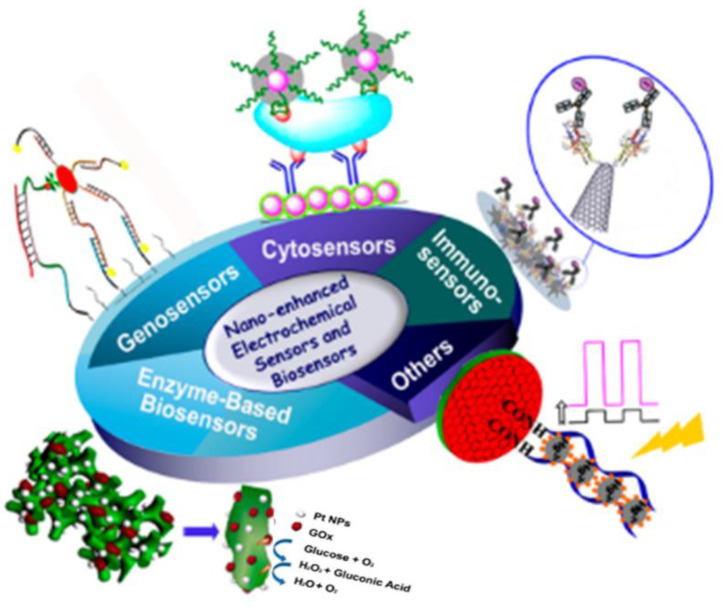
Schematic of electrochemical sensors and biosensors related to nanomaterials and nanostructures, in which electrochemical sensors for small molecular, enzyme-based biosensors, genosensors, immunosensors, and cytosensors are demonstrated. Reprinted with permission from Zhu, C.; Yang, G.; Li, H.; Du, D.; Lin, Y. Electrochemical sensors and biosensors based on nanomaterials and nanostructures. Anal. Chem. 2015, 87, 230–249. American Chemical Society.

PANI attracts extensive attention as a sensor due to its different structures with different morphologies, such as nanowires (Figure 27). Various kinds of precision sensors, such as chemical and biological sensors, will be produced by PANI [189]. Researchers have tried to use PANI with different nanostructures in gas sensors, such as nanofibers, nanowires, and nanotubes, due to its extraordinary surface area and gas emission potential. Gas interacts with PANI and is immersed chemically and physically [190]. It is used to detect H_2_S. H_2_S cannot change the conductivity of PANI due to its low acidity, so gold nanoparticles precipitate to increase the sensitivity of PANI. It responds to low concentrations up to 0.1ppb with good reproducibility [191]. PANI nanofibers have been used to identify ammonia, hydrazine, hydrochloric acid, methanol, and chloroform, resulting in swelling, doping, reduction, and a structural change in PANI nanofibers. In addition, exposure time shows the main effect on detection aptitude in the resulting methods [192]. Methanol does not affect the oxidation state and reduces doping, but it will swell the PANI chains and cause a change in resistance. The use of camphor sulfonic acid (CSA), such as a drug, raises the response of PANI to alcohol fumes [193]. Alcohols, because of their chain size, mark the resistance electrical of PANI and results in poly(N-ethyl aniline), poly(N-methyl aniline), and poly(diphenylamine) [194]. Hydrogen will interact with the amine nitrogen position of the doped PANI in hydrogen sensors that touches the conductivity of PANI [195]. NH_3_ is easily distinguishable from alcohols due to its high electron affinity. The TiO_2_ sensors applicable to detect NH_3_ require extraordinary temperatures or platinum doping; nevertheless, TiO_2_/PANI sensor facilitates detection at ambient temperature [188]. Chlorinated and nitrogen dioxide hydrocarbons were discovered by Cu-PANI and Au-PANI, respectively [196]. As a result of its environmental stability, biocompatibility, and electron transfer arbitration of oxidation or enzymatic reduction reactions, PANI has been utilized as a biosensor. Glucose is an essential substance in the body and can cause diabetes [199]. Consequently, glucose control is the main problem in the structure of PANI for biosensors [197]. PANI-based biosensors will be classified into several clusters that involve DNA and safety sensors. PANI possesses two or three redox oxidation modes that facilitate enzyme–polymer charge transfer and acts as an electron transfer medium. Hence, usage of other intermediates of electron transfer will be reduced [200]. Enzyme-based sensors can lead to enzyme feedback at the surface electrode for competence and detection; they are known as oxidation reductives [200].

PANI can be used as sensors utilized as an electronic tongue, involving six or seven sensors to detect taste as effectively as human taste receptors, to develop produce value in the food business. This organic procedure is proportional to sensors generating electrical signals, such as potentiometric changes. The integration of information with the sensors makes a ‘fingerprint’ of every taste. Hydrogen ions can detect acidity in citric acid and hydrogen chloride, whereas sodium chloride and sugar indicate salt and sweetness, respectively [246].

Magnesium chloride identifies the bitterness of chemical composites, such as caffeine. Disodium guanylate and monosodium glutamate (MSG) are responsible for the umami (taste) in meat/fish and seaweed, respectively. The oligomer of aniline and polypyrrole are placed on integrated electrodes to detect saline, bitter, sweet, and acidic dilution in mineral water, coffee, and tea [201]. Gold-modified microelectrodes are changed by polyamide. Electrospun nanofibers of PANI are similarly studied to identify tetracycline residues in fat and skim milk samples [247]. AgCl-PANI has also been designed and used to detect salt, acid, and emulsion solutions [202].

#### 6.1.5. Supercapacitors

The technological development of humans and the importance of energy have drawn attention to efficient energy storage and consumption in recent years. Supercapacitors, fuel cells, and lithium–ion batteries are the most important energy storage devices in the field [204,205]. High-capacity capacitors have been filled the gap between electrolytic capacitors and rechargeable batteries. The capability of energy storage for an electrolyte capacitor will be 10–100 times lower than a supercapacitor, so supercapacitors have additional profits compared with conventional capacitors. Furthermore, supercapacitors carry charges faster than batteries; they are more durable in charge and evacuation cycles. Supercapacitors can be divided into three main clusters: (1) double-layer electrostatic capacitors related to carbon electrodes; (2) electrochemical pseudocapacitors related to conducting polymer electrodes such as metal oxide; (3) hybrid pseudocapacitors, which are a combination of the two previous ones [206].

**Figure 28:**
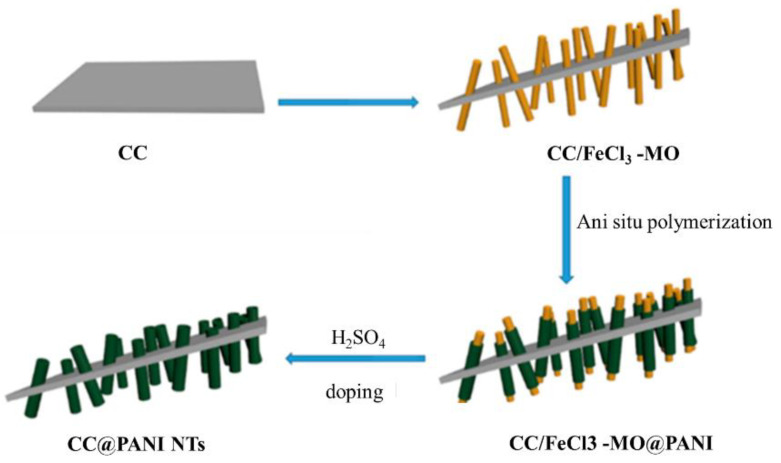
Schematic of the fabrication route of CC@PANI NTs.

Electrode materials have an important impact on the efficacy of the storage device and energy conversion. Electrodes of storage devices are mainly related to conductive polymers, metals, and carbon-based materials [207]. Although carbon-based materials show extraordinary conductivity, power density, and adequate durability, their energy density is usually low. Metals show adequate electrochemical characteristics, but their cost and lack of natural abundance limit their use. PANI, because of its high conductivity, various oxidation states, and high specific capacitance, can be used as a supercapacitor. Hence, the conductivity and flexible electrochemical properties of PANI make it highly suitable for capacitor manufacturing. The properties of PANI-based supercapacitors are highly dependent on synthesis, doping, physical/chemical features, and nanostructure. From 0.8 V to 1 V, PANI is subjected to a proper charge/discharge process; though, at less than 0.6 V, PANI is unworkable due to the minimum density of energy [208]. PANI is joined by the carbon group, such as carbon nanotubes and graphene, to be utilized in supercapacitors (Figure 28) [78]. The polymerization for PANI graphene oxide produces homogenous PANI nanofibers that demonstrate extraordinary specificity, conductivity, and capacity but adequate cyclic stability [209].

### 6.2. Medical Applications

**Figure 29:**
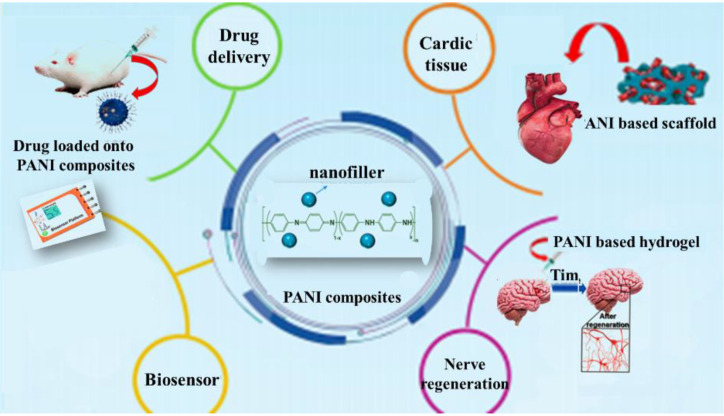
Schematic of PANI in medical applications. Reprinted with permission from Zare, E.N.; Makvandi, P.; Ashtari, B.; Rossi, F.; Motahari, A.; Perale, G. Progress in Conductive Polyaniline-Based Nano-composites for Biomedical Applications: A Review. J. Med. Chem. 2020, 63, 1–22, doi:10.1021/acs.jmedchem.9b00803. American Chemical Society.

PANI, as one of the most familiar ICPs, has significant potential applications in biomedicine due to its high electrical conductivity and biocompatibility caused by its hydrophilic environment, low toxicity, high environmental stability, and nanostructured morphology. This review explains the state-of-the-art biological activities and applications of PANI-based nanocomposites in the medical fields, such as neural prosthesis/biotic–abiotic interfaces, scaffolds, and delivery systems (Figure 29) [248].

#### 6.2.1. Neural Prosthesis/Biotic–Abiotic Interfaces

People with central nervous structure syndromes suffer from the inability to communicate, move, and control their environment. Brain–computer edge (BCI)/brain–machine interface (BMI) via neural prostheses can be considered to compensate for such weaknesses [211]. Cochlear implants are devices in which the acoustic nerves are stimulated over arrays of microelectrodes. The neural electrodes, neural tissue, and bioelectric signals are transduced to electrical signs with the neural prosthesis [212]. The connection between neural tissue and neural electrodes can be a hot spot for technical challenges, such as modulus, biocompatibility, and conductivity [213]. Minimum impedance electrodes possess advantages due to their ability to decrease signal-to-noise fraction and increase the recording value during the progress of stimulus [214]. Conventional electrodes show extraordinary impedance (e.g., large useful potential origins and a damaging electrochemical reaction) because of the small surface area; therefore, nanostructures are employed in neural prosthetic uses due to their maximum surface area and minimum impedance [192]. Conductive nanostructure polymers reduce impedance electrode but considerably improve charge transmission capability [192].

The charge density of the human retina is around 0.08–1.91 mC/cm^2^, which is necessary to generate neuronal activity during stimulation. Electrochemical impedance spectroscopy characterizes the neuronal interface [215]. PANI covered with Pt electrode can be utilized as a neural probe. A modified electrode shows little resemblance to the uptake of essential retinal components and inactivity along with lipid peroxidation compared with the original electrode [210]. Electrical stimulation increases the uptake of human plasma fibronectin (FN) and bovine serum albumin (BSA). The accumulation of BSA stimulation institutions and the PANI nanostructure may prevent this phenomenon [216]. Scarring and inflammation occur with long-term electrode implantation [217]. Fragments of the retina are likely to aggregate on the modified surface of the electrode, leading to inflammation and diminished scarring. The modified electrode demonstrates suitable biocompatibility in long-term implantation, while the original electrode experienced corrosion; therefore, PANI performance is poor in terms of shielding membranes and anti-eroding purposes on the Pt electrodes. The adapted electrode shows the opposite biocompatibility in long-term implantation [210]. PANI is changed from a hydrophilic to hydrophobic material under doping. It recovers hydrophobicity under a redox action, and the morphology for PANI becomes heterogeneous [218]. To compensate for the discrepancy between the modulus of the probe and the modulus of the tissue, a conductive polymer is prepared in the hydrogel to provide a soft electrode. The surface area of the conductive polymer will increase because of the large area in the hydrogel framework. Therefore, the electrode impedance declines the transport of signals through the neurons [223].

#### 6.2.2. Scaffolds

**Figure 30:**
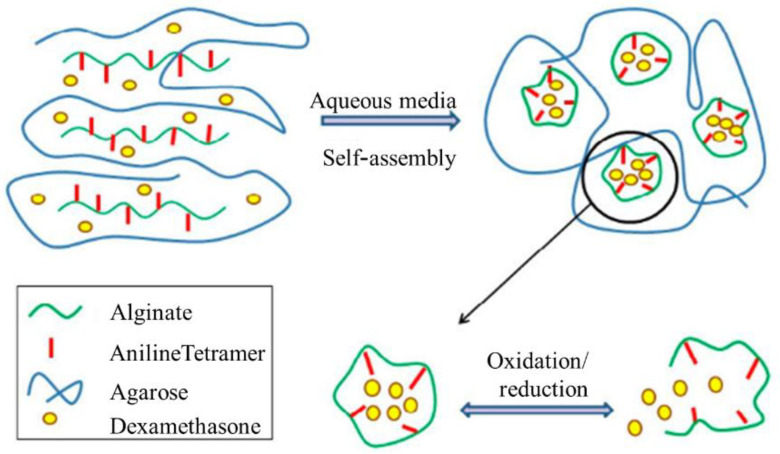
Schematic of drug release: ADA-AT chains self-assemble into vesicles in an aqueous medium, and dexamethasone molecules are entrapped in them. By applying electrical potential, vesicles are disrupted during oxidation/reduction procedures, leading to drug release. Reprinted with permission from Atoufi, Z.; Zarrintaj, P.; Motlagh, G.H.; Amiri, A.; Bagher, Z.; Kamrava, S.K. A novel bio electro active alginate-aniline tetramer/ agarose scaffold for tissue engineering: synthesis, characterization, drug release and cell culture study. J. Biomater. Sci. Polym. Ed. 2017, 28, 1617–1638.Taylor & Francis.

Currently, the global quality of life is on an upward trajectory because of the invention of synthetic structures. Tissue engineering can play a key role in the development of unique materials to duplicate the functions of organs. The biomimetic performance of the materials implanted is a fundamental factor in the creation of new substrates in the body. Tissue engineering involves three parts: cells, drug growth factors, and scaffolds [224]. Scaffolding is the most significant element for tissue engineering because it mimics the conditions of the organ and allows the transport of drugs, thereby resulting in cells regenerating damaged tissue (Figure 30) [227]. Implanted scaffolds should be biocompatible to reduce the risk of rejection in the body [229]. The architecture of the scaffold has been performed for the proposed organs. Normally, organs show better compatibility by conductive substrates due to cellular potentials, but compatibility changes from organ to organ [226]. For instance, cardiac and nervous tissues act as potential tissues that require conductive scaffold usage. Conductive polymers present different categories in tissue engineering for scaffolds. PANI and its products possess antioxidant, antibacterial, biocompatibility, and hemostatic characteristics necessary for any given scaffold; consequently, PANI and its products have been used in tissue engineering and regenerative medicine [227,228].

Because of their biodegradation characteristics, oligomers of aniline attract a lot of consideration for their usage as scaffolds in the body. In addition, they had presented adequate interaction by dissimilar forms for tissues, i.e., cells, such as skinning [230]. For instance, agarose scaffolds or oligoaniline-alginate have shown suitable adhesion then proliferation for PC1_2_ cells [227]. Furthermore, it was shown that the Schwann cell myelin gene expressions were considered more frequently of the electrical characteristics of the substrate than the modulus. Myelination of Schwann cells is an essential factor in the regeneration of peripheral nerves; similar to conductive polymers that are related to aniline pentamer, the high secretion of neurotrophins from Schwann cells promotes neural regeneration [228]. Injectable, conductive, and self-regenerating hydrogel related to PANI-chitosan is used for wound dressings because of its antioxidant, hemostatic features [221]. Myocardial infarction (MI) is a heart disorder characterized by cardiac dysfunction. Therapy cells can be one approach to heart tissue regeneration. Conductive, injectable, and self-regenerating hydrogel related to the chitosan–aniline tetramer by antimicrobial activity can be utilized, such as the cellular release carrier of cardiac regeneration [231]. Hence, ductile hyperbranched aniline–polylactide tetramer can be utilized in myoblast differentiation for the purpose of strength tissue engineering [219]. In general, conductive polymers affect the morphology of cells and aid in their growth, proliferation, and differentiation [222].

#### 6.2.3. Delivery Systems

The biggest challenge in the drug delivery method is smart and organized drug delivery. The drug release depends on the situation of its environment, such as pH and temperature [227]. As a result of their electroactive nature, conductive polymers are stimulated with external conditions, such as electric current/field; drugs can then be released based on arbitrary conditions. These polymers will be charged and then discharged through a decreased state [232]. Protein adsorption is linked to the redox state, and electrical stimulation improves adsorption [233].

**Figure 31:**
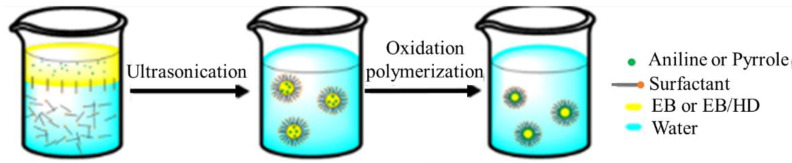
Oxidative polymerization of aniline or pyrrole in miniemulsion to form conductive polymer nanocapsules in the existence of ethylbenzene (EB) or ethylbenzene/hexadecane (EB/HD). Reprinted with permission from Lv, L.-P.; Zhao, Y.; Vilbrandt, N.; Gallei, M.; Vimalanandan, A.; Rohwerder, M.; Landfester, K.; Crespy, D. Redox Responsive Release of Hydrophobic Self-Healing Agents from Polyaniline Capsules. J. Am. Chem. Soc. 2013, 135, 14198–14205, doi:10.1021/ja405279t. American Chemical Society.

Scaffolds can be versatile vehicles for delivering proteins, genes, and hydrophobic/hydrophilic drugs. Hydrophobic drugs show rapid discharge from a hydrophilic scaffold; therefore, the inclusion of the drug in an appropriate vehicle is necessary. PANI-based scaffolds generally comprise mostly hydrophilic biocompatible polymers and hydrophobic drugs that become tricked by PANI in aqueous media (Figure 31). The formation of nanoparticles can release a profile for controlling features with electrical stimulation [227]. Vesicle assembly splits under oxidizing voltage by eliminating the voltage. Hence, it demonstrates a revocable and controllable process [234]. Drug release will be used with polarity variant or segmental cleavage by voltage difference, and it is devoid of chemicals. Administration of non-viral DNA is the best method of DNA transport; for example, polyethyleneimine (PEI) can be used as a cationic polymer. The PEI-aniline tetramer has been suggested as a genetic material therapy in reformative medicine. As a result of electrostatic repulsion in aqueous media, it is shaped like a core-shell structure by the core of aniline tetramer via self-assembly [235].

### 6.3. Anti-Corrosion Material Applications

Corrosion generates many expenses for companies in the industrial world. A realistic way to address this problem is surface coating. Conductive polymers keep substrates, such as iron, in the passive state and act as an irritable film due to their extraordinary redox potential. Decreasing oxygen dissolved in a medium acid is affected by the catalytic effect caused by a conducting polymer. At the beginning of coating, the metal oxide film can prevent oxidization because of the electrochemical procedure, in which the conductive polymer oxidizes the surface of the metal. In addition, the conductive polymer shows a facilitator between solution and metal; the oxidized conductive polymer is produced in the beginning with the oxidation method [237].

**Figure 32:**
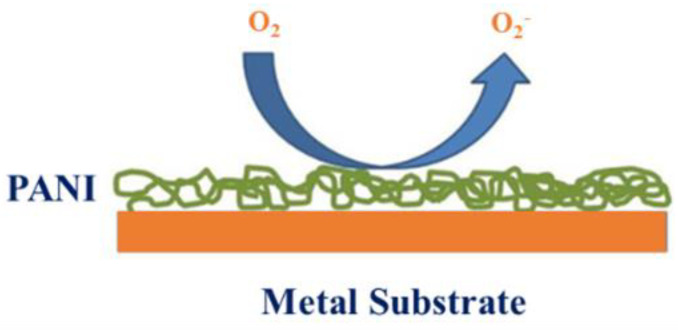
Mechanical image of the metal surface protected by PANI coatings against corrosion. Reprinted from Fundamentals and Emerging Applications of Polyaniline, Zarrintaj, P.; Vahabi, H.; Saeb, M.R.; Mozafari, M, Application of polyaniline and its derivatives, pp. 259–272., Copyright (2019), with permission from Elsevier.

PANI is utilized to protect iron (Figure 32) [161], and it compares favorably with conventional polymers such as polyvinyl chloride [238]. PANI acts as a suitable coating in acid solutions. Hence, PANI was found to protect stainless steel in NaCl media poorly. PANI’s shielding with sulfuric acid is superior to HCl [239].

**Figure 33:**
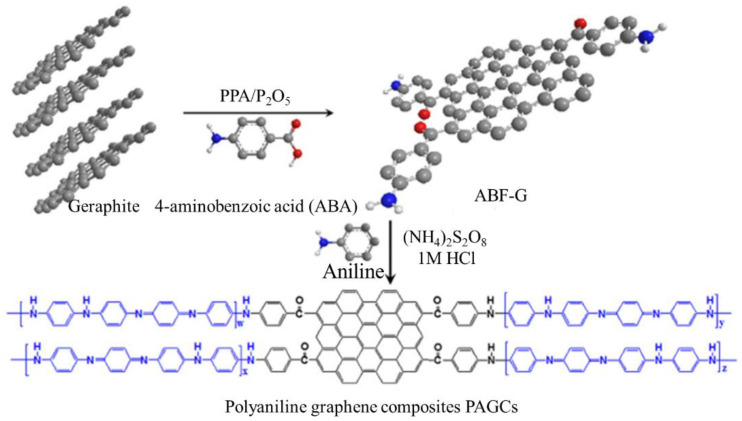
Preparation of PANI/graphene composites (PAGCs). Reprinted from Carbon N. Y., 50 Chang, C.-H.; Huang, T.-C.; Peng, C.-W.; Yeh, T.-C.; Lu, H.-I.; Hung, W.-I.; Weng, C.-J.; Yang, T.-I.; Yeh, J.-M., Novel anti-corrosion coatings prepared from polyaniline/graphene composites, pp. 5044–5051., Copyright (2012), with permission from Elsevier.

The nanocomposite of PANI/graphene demonstrates brilliant anti-corrosion properties in contrast to O_2_ and H_2_O, and it is even superior to pristine PANI and PANI–clay composite; its remarkable surface area for properly dispersed functionalized graphene in the polymer matrix results in improved diffusion, which postpones corrosion (Figure 33) [236]. Carbon steel covered with rubber-modified polybenzoxazine (PBZ) has been utilized as a vehicle of the emerald salt of PANI. PBZ has minimum surface free energy, minimum shrinkage, minimum water absorption, and adequate thermal/mechanical characteristics. PBZ exclusively shields steel, but its compound by PANI exhibits better protection characteristics than pristine rubber. The need for PANI is obvious in long-term exposure [240].

## 7. Conclusions

This review is a comprehensive overview of PANI and PANI thin films with several preparation methods and properties, highlighting the advantages of different applications. Several non-conventional polymerization methods have been employed to synthesize PANI and are extensively used by dissimilar oxidants and dopants. However, the most common method for the preparation of thin films of PANI is oxidative polymerization. Likewise, thin films of PANI can be prepared via polymerization of surface-initiated electron method, Langmuir–Blodgett method, polymerization of atmospheric pressure plasma method, and mSILAR method. These novel synthetic approaches allow PANI to show different physiochemical properties and facilitate control of high electrical conductivity, environmental stability, and thermal stability. Given that PANI shows various properties such as magnetic features, electrical and dielectric properties, charge–discharge behavior, redox features, antioxidant properties, anti-corrosion features, capacitive properties, and sensing features, it is suitable in several fields for numerous applications. Such applications include electrochromic glass, LED production with electroluminescent properties, solar cell production technology due to low-cost features, PANI-based sensors such as gas and glucose sensors for heavy metal detection, and supercapacitors because of various oxidation. In addition, PANI applications in medicine involve engineering, and advances in this area require new and intelligent equipment. Neuroscientists require devices that improve nerve weakness and contribute to the development of neuroscience. Scaffolding has been utilized to correct organ disorders, and biocompatible conductive scaffolding shows appropriate biocompatibility properties. As a result, PANI utilized in delivery systems has attracted a great deal of attention, resulting in innovative delivery systems, such as electro-drug delivery systems. Conductive PANI-based nanocomposites represent a new, but not yet completely explored, area of quantum dots where promising results and unresolved technology challenges both call for opportunities for deeper studies and developed research. There are some major limitations, such as processability and physicochemical properties on PANI applications and its nanocomposites into sensor practice. PANI is gaining increasing interest from the scientific community. However, from an industrial perspective, some of the challenges have important drawbacks on regulatory and quality assurance issues, thereby explaining the relatively low amount of sensor data related to PANI. Lastly, the use of PANI based on quantum dots as a sensor barrier was studied and performed successfully.

## Figures and Tables

**Table 1:** Modes of electrodeposition.

Mode of Electrodeposition	Difference
Galvanostatic	Deposition by applying a constant current between the counter and working electrodes
Potentiodynamic	Electrode potential is varied using a stable reference electrode, and the current flow is measured between the working and counter electrode
Potentiostatic	Deposition by applying a constant potential between working and counter electrodes

**Table 2:** PANI nanocomposites used in several applications.

Sensing Materials	Preparation Method	Features	Application	References
Polyaniline-based pseudocapacitive glass	Facile thermal evaporation and electrodeposition methods	Excellent energy—storage and electrochromic features	Electrochromic glasses	[162,163,164,165,166,167,168,169,170,171,172,173,174]
Polyaniline-SO_4_^2−^, BF_4_^−^, CL^−^, ClO4^−^, and p-toluene sulfonate (TsO^−^)	In situ electropolymerization	Easy synthesis, low price, and good conductivity features	Solar cells	[175,176,177,178,179,180,181,182,183]
Polyaniline-poly(styrene sulfonate) (PANI- PSS)	Chemical oxidation polymerization	An environmentally benign route for processing-controlled doping with various water-soluble polymeric or molecular dopants	Electroluminescence machines	[184,185,186,187]
Polyaniline (PANI)–Titanium dioxide (TiO_2_) ammonia gas sensors	Polymerization, spin-coating method on glass	Different structures with different morphologies, such as nanowire features	Sensors	[188,189,190,191,192,193,194,195,196,197]
Polyaniline, bovine viral diarrhea virus	Oxidative polymerization	Electronic and bio-molecular features	Biosensor	[198,199,200,201,202]
Graphene/polyaniline composites	Hydrothermal synthesis	High-capacitance electrode material feature	Supercapacitors	[78,203,204,205,206,207,208,209]
PANI-coated platinum (Pt) electrode	In situ polymerization	Excellent intactness and the stable nanoparticle morphology features	Neural prosthesis/biotic–abiotic interfaces	[210,211,212,213,214,215,216,217,218]
PU-PANI nanofibrous scaffolds	Electrospinning method	Considerable electrical conductivity, biocompatibility, and ease of synthesis	Scaffolds	[219,220,221,222,223,224,225,226,227,228,229,230]
Agarose/alginate-aniline tetramer	Oxidative polymerization	Possessing electroactive nature feature	Delivery systems	[231,232,233,234,235]
Polyaniline/graphene composites	Chemical oxidation polymerization	Outstanding barrier features against O_2_ and H_2_O compared with neat polyaniline and polyaniline/clay composites (packs)	Anti-corrosion material applications	[236,237,238,239,240]
Polyaniline nano-membranes	In situ polymerization	High chemical stability feature	Gas separation membranes	[9,38,192,241]
Polyaniline (PANI) nanoparticles coated by nanolayer of bismuth oxide Bi_2_O_3_	Polymerization	Possess functional groups and good structural features	Photovoltaic cells	[242,243]

## Data Availability

The data presented in this study are available on request from the corresponding author.
